# Preliminary efficacy of aerobic training among university students with migraine symptoms: Study protocol for a pilot randomized controlled trial

**DOI:** 10.1371/journal.pone.0291534

**Published:** 2023-09-25

**Authors:** Kiruthika Selvakumar, Tan Lee Fan, Foo Chai Nien, Mun Hou Kit

**Affiliations:** 1 M. Kandiah Faculty of Medicine and Health Sciences, Department of Physiotherapy, Universiti Tunku Abdul Rahman, Selangor, Malaysia; 2 Department of Mechatronics and BioMedical Engineering, Lee Kong Chian Faculty of Engineering and Science, Universiti Tunku Abdul Rahman, Selangor, Malaysia; 3 M. Kandiah Faculty of Medicine and Health Sciences, Department of Population Medicine, Universiti Tunku Abdul Rahman, Selangor, Malaysia; Hamasaki Clinic, JAPAN

## Abstract

**Background:**

Migraine is a primary neurological headache. Treatment of this condition includes medications; however, these medications, when given for a longer duration, can have side effects. If migraine is left untreated or undiagnosed, it is reported that around 2.5% of individuals with migraine may develop to have a chronic condition. This study aims to analyse the preliminary effectiveness of aerobic training on migraine pain level, sleep quality, quality of life, and resting-state brain waves among university students with migraine symptoms.

**Methodology:**

88 university students with migraine symptoms are the target participants. 4 of 5 on the Migraine Screen Questionnaire, 5 of 7 on the International Classification of Headache Disorders 3rd edition (ICHD-3), and both genders aged 18–40 years will be included. The participants with a score of more than or equal to 5 on the visual aura rating scale, diagnosed with a secondary headache, pregnancy, medication for neurological and cardiorespiratory conditions, and unwilling to participate will be excluded. Based on the disability questionnaire, the participants will be randomly assigned to either of the three groups. The primary outcome is resting-state electroencephalography (EEG) brain, and the secondary outcomes are sleep quality, quality of life, and migraine pain level. The post-test assessments will be performed at week 6.

**Result:**

After the primary EEG analysis using MATLAB, the amplitude, frequency, frequency band ratio, and power spectrum density will be analysed. Mixed design analysis and intention-to-treat analysis will be used to assess the efficacy of aerobic training.

**Discussion:**

Migraines can be unpredictable, sometimes occurring without symptoms. If underdiagnosed or over-looked, it encompasses a serious of long-term effects. Hence with appropriate intervention, the symptoms can be prevented from worsening. But there is an unmet need for evidence-based non-pharmacological approaches to complement pharmacotherapy in migraine prevention. Moreover, an exercise intervention may be more suitable for people with migraine considering their tendency toward inactivity. Although some studies developed exercise programs for untrained patients with migraine, the outcome was primarily in terms of exercise capacity rather than the primary characteristics and secondary brain wave/ sleep quality changes, indicating the need for this study.

## Background

Migraine is a primary neurologic headache [1], often accompanied by nausea, vomiting, photophobia, phonophobia, or vertigo, and may present with or without aura [2]. It is a prevalent disorder, affecting 15.1% of the world’s population [3]. It is a leading cause of disability worldwide; approximately half of those affected have such severe attacks that they cannot function normally in routine daily activities [4]. It also results in loss of quality of life (QoL) as well as having a significant impact on society as a whole [5]. Treatment of this condition includes medications such as beta-blockers, anticonvulsants, calcium channel blockers, tricyclic anti-depressants, and non-steroidal anti-inflammatory drugs [6]. Specifically, for abortive treatment, non-steroidal anti-inflammatory drugs (NSAIDs) are mainstay choices and have the greatest strength of evidence, followed by triptans, antiemetics, and ergotamines. The drugs of choice for preventive treatment are beta-blockers, antiepileptics, calcium channel blockers, and anti-depressants [1]. However, these medications, when given for a longer duration, can have side effects like constipation, cardiac conduction defects at higher doses, dizziness, flushing etc, depending on the medication used [1]. Additionally, recent evidence supports anti-CGRP antibodies for migraine, but the reported side effects include injection-site pain, pruritus, and erythema [7]. And also, currently, the headache disorder being a bio-psychosocial phenomenon, pharmacotherapy fails to address the psychological and social factors [5].

There are several hypotheses for migraine generation. The proposed mechanisms being: vasodilation of cerebral vasculature structure [8], drug-induced [9], hyperexcitability in cortical and brainstem areas [10,11], cortical spreading depression are responsible for migraine [12], and evidence also show trigeminal afferents projecting to meninges releasing pain mediators like substance P, calcitonin gene-related peptide, and neurokinin [13]. Occasionally, the pain is also due to hypertrophic nasal turbinates or deviated nasal septum irritating branches of trigeminal nerves [14].

The treatment for primary headaches is not common, it depends on severity, symptoms, and disability. Although medications were a common treatment choice, according to the Migraine research foundation, there are 3 main types of non-drug treatments for migraine. They are lifestyle advice, therapies, and exercises. Lifestyle advice includes seeing a doctor, maintaining a headache diary, sticking to the same eating and sleeping schedule [15]. Therapies include physical therapy, acupuncture, yoga, tai chi, massage, stress management, biofeedback, hypnotherapy, and cognitive behavioural therapy [16]. Especially, the EMG biofeedback has a beneficial effect on autonomous nervous system activity, renders individuals resilient to stressors, and possibly mediates a beneficial afferent vagus nerve stimulation. Through regular training, individuals experience a long-lasting reduction in muscle tension, a rise in peripheral skin temperature, and a lower heart rate [25], suggesting a feasible and cost-effective treatment option. Similarly, exercise programs are also frequently recommended to promote health, and the general guidelines include low-impact activities like tai-chi, yoga, isometrics, or band exercise [17]. Enkephalin, a natural anti-depressant released during exercise, aids in lowering stress levels, which is thought to be a primary migraine trigger. Therefore, to lessen the burden of daily medication intake, migraine patients can embrace exercise as a prophylactic or preventive treatment. Regular aerobic exercise, in particular, can help migraine sufferers by enhancing sleep quality, assisting those who are overweight or obese in losing weight, and lowering cardiovascular risk factors. Studies also found that exercise treatment significantly increased maximal oxygen uptake compared to the other treatments [18]. Hence both EMG biofeedback and exercises focus on the ability of the individual to relax during a stressful event, thereby improving physical fitness. Non-invasive neuromodulation constitutes a valuable approach with strong backing evidence, particularly in the case of sTMS and transcutaneous cranial nerve stimulation [19]. Behavioural treatment approaches can be considered an add-on to ongoing treatments and often offer a positive clinical improvement [20]. The use of non-pharmacological treatments for migraine represents an expanding clinical practice and an interesting area of research [19].

Habitual aerobic exercise has a major advantage of preventing or reducing symptoms of several chronic diseases and medical conditions [21]. Aerobic or cardiovascular exercise is a form of bodily movement fueled by aerobic energy-generating processes, where the energy demands of the exercise do not exceed the rate at which the cardiovascular system can supply oxygen to working muscles [22]. Aerobic exercises have already been proven to reduce the frequency, duration, severity or associated disability in migraine [21]. The physiology is that when one exercise, the body releases endorphins, the body’s natural painkillers and natural anti-depressants chemicals called enkephalins. According to Centre of Disease Control and Prevention (CDC), adults should exercise 150 minutes of moderate-intensity aerobic exercise and two or more days a week of muscle strengthening each week to relieve migraine or primary headaches. Similarly, a study comparing neck treatment and aerobic exercise concluded that five weeks of intervention for migraine patients responded with better outcomes [23]. According to the American migraine foundation, the exercise program should include cardiorespiratory fitness, flexibility exercises, and muscular strengthening. For migraines, mild to moderate aerobic exercise is highly recommended. But most of the studies’ primary outcome is only pain intensity, severity, duration, and frequency. Recent research found that the resting state brain waves were altered during the neuroimaging [24], and that the spatio-temporal dynamics exist in migraine patients during the resting-state. Moreover, the temporal dynamics, spatial changes, and clinical traits such as migraine disability interact [25], indicating the need for further analysis and appropriate treatment strategies. Hence this study aims to assess the preliminary effects of aerobic training on migraine pain level, sleep quality, quality of life, and resting-state brain waves compared with EMG biofeedback training and placebo group.

### Study goal and objective

If an earlier, migraine is left untreated or undiagnosed, it is reported that around 2.5% of individuals with migraine are considered to have a chronic condition, defined as presenting with more than 15 headache days within a month. Subclassifications of the disease (migraine with and without aura and chronic migraine) occur clinically at various severity levels and coexist with numerous comorbidities. For this reason, several studies encourage a multidisciplinary and tailored treatment approach for these patients [26,27]. This preliminary randomized control trial aims to analyse the effectiveness of aerobic training among university students with migraine symptoms. The main objective is to determine the preliminary effect of aerobic training on migraine pain level, sleep quality, quality of life, and resting-state brain waves compared with EMG biofeedback training and placebo.

The alternative hypothesis of this study will be the aerobic training will be superior to EMG biofeedback training and placebo group in improving migraine pain level, sleep quality, quality of life, and resting-state brain waves. The null hypothesis will be the aerobic training is same as to EMG biofeedback training and placebo group in improving migraine pain level, sleep quality, quality of life, and resting-state brain waves. Although the Migraine Research Foundation listed three main types of non-drug treatments for migraine: lifestyle advice, therapies, and exercises. Some common aerobic exercises such as walking, jogging, a behavioral weight loss program, cycling, and a combination of cross-training, walking, jogging, and cycling are suggested to be beneficial to migraine patients but there remains no specific protocol established till now. Hence, this study will contribute to the research field by establishing an aerobic training protocol for patients with migraine symptoms.

## Methodology

This study is a pilot randomized control trial with a pre-and post-test design that focuses on the preliminary efficacy of aerobic training among university students with migraine symptoms. The primary endpoint is resting-state electroencephalography (EEG). The secondary endpoints are sleep quality, quality of life, and migraine pain level. This study will be conducted at Physiotherapy Center, Universiti Tunku Abdul Rahman, Sungai Long Campus. The outcome measures will be performed at baseline and end of intervention (6^th^ week) to compare the data obtained. The study is approved by UTAR Scientific and Ethical Review Committee (U/SERC/188/2022). The informed consent form will be signed and dated before the start of the actual intervention. Parallel assignment intervention model will be used. The study is also registered with ClinicalTrials.gov Protocol Registration and Results System (Identifier: NCT05741775).

### Inclusion and exclusion criteria

The target participants for this research will be undergraduate and postgraduate students from Universiti Tunku Abdul Rahman. The participants who fulfil the following criteria will be recruited for the interventional phase. 4 of 5 on the Migraine Screen Questionnaire, and 5 of 7 on International Classification of Headache Disorders 3rd edition (ICHD-3). Both genders aged between 18–40 years. The participants with a score of more than or equal to 5 on the visual aura rating scale, diagnosed to have a secondary headache (headache attributed to the causative disorder example: infection, trauma, or injury to head/ or neck) [28], pregnancy, took medication for neurological conditions like stroke, multiple sclerosis and took medications for cardiorespiratory conditions like asthma, took medications for headache and unwilling to participate will be excluded.

### Sample size

Sample size is calculated based on migraine, biofeedback training [29] and aerobic training articles [30]. Using g power statistical tool F test, ANOVA: repeated measure, between factors, and with 0.4 effect size, 0.05 alpha error, 0.95 beta error, 3 groups, 4 outcome measurements the sample size is 66. Then, further adjustment of sample size to accommodate 25% dropout rate [31] gives the final estimated value to 88.

### Randomization

A simple random sampling method will be employed as the sampling technique in recruiting the subjects. Upon successful recruitment of participants, and with Migraine Disability Assessment Scale (MIDAS) Questionnaire, the subjects will be allocated into either of the three groups (Group 1: aerobic training, Group 2: biofeedback training, Group 3: placebo group). The subjects will be given a random number in an excel sheet and ranked. Following ranking, the ranked participant will be divided by size and group accordingly.

Simultaneously using MIDAS, each group will have 7–10 participants depending on each level of disability on MIDAS. In the Migraine Disability Assessment Scale (MIDAS) Questionnaire that assesses headache-related disability, the respondents are required to answer five questions. The number of days in each question will be summed up and the scoring indicates the activity limitation due to migraine in the past 3 months. The participants are then categorized into no or little disability (0–5), mild disability (6–10), moderate disability (11–20), and severe disability (21+) (Stewart et al., 2001).

### Blinding

A single blinding method will be used. The outcome assessor will be blinded. The outcome assessor will not know whether participants are from aerobic training, biofeedback training or placebo group. Single blinding will be used in this study because the results are less likely to be biased.

### Eligibility screening questionnaire

The Migraine screen questionnaire (MS-Q) is a five-item migraine screening questionnaire developed for use in clinical practice. The questionnaire is based on the international headache society criteria (IHS) on migraine diagnosis [32]. Each of the five items is a structured questionnaire that has an option of yes/no. A score of 0 is assigned for each “NO” response and 1 for each “YES” response. The total score is 5, where a cut-off point of ≥4 was used to indicate a case of migraine [33].

Visual Aura Rating Scale (VARS) is the weighted sum of the presence of five visual symptom characteristics: duration 5–60 min (3 points), develops gradually ≤5 min (2 points), scotoma (2 points), zig-zag lines (2 points), and unilateral (1 point). The maximum score is 10 points. A VARS score of 5 or more diagnosed migraine with aura [34]. The newly validated instrument to assess COVID-19 among migraine participants will also be used to exclude the other secondary headache.

Additionally, the International Classification of Headache Disorders 3^rd^ edition (ICHD-3) criteria will be evaluated, and it suggests at least five of seven attacks fulfilling the criteria. [35]. Migraine may be diagnosed without aura with the following diagnostic criteria:

1. 5+ attacks fulfilling the other criteria
2. Headache attacks that last from 4 to 72 hours (untreated or unsuccessfully treated)
3. Headache consisting of at least 2 of the following characteristics: unilateral location, pulsating quality, moderate/severe pain intensity, and aggravation by or causing avoidance of routine physical activity (i.e., walking or climbing stairs)
4. During the headache, the presence of at least one of the following: nausea/vomiting, photophobia/phonophobia
5. Not better accounted for with another ICHD-3 diagnosis

### Outcomes

The primary endpoint is resting-state EEG, and the secondary endpoints are sleep quality, quality of life, and migraine pain level. The Confounders will be the level of migraine pain (Russo et al., 2018), gender (Victor et al., 2010) (Kelman, 2006), and age (Echiverri et al., 2018), COVID-19 (Haghdoost et al., 2021).

The migraine pain level questionnaire that records the general health condition of the participants and the characteristics of migraine which including, frequency, severity, and duration will be included. Level of pain scored on a four-point numerical rating scale (0–3) equivalent to no, mild, moderate, and severe pain [36]: 0 no pain. 1 mild pain, does not interfere with usual activities 2 moderate pain, inhibits but does not wholly prevent usual activities 3 severe pain, prevents all activities. The other components are expressed as either decreased/ increased/ remains the same/ unable to recall.

Pittsburgh Sleep Index is a self-rated questionnaire that assesses sleep quality and disturbances over a 1-month interval. 19 individual items generate seven component scores: subjective sleep quality, sleep latency, duration, habitual sleep efficiency, sleep disturbances, use of sleeping medication, and daytime dysfunction. The sum of scores for these seven components yields one global score. The sleep component scores are summed to yield a total score ranging from 0 to 21, with the higher total score (referred to as global score) indicating worse sleep quality [37].

Migraine Specific Quality of life is a 14-item instrument that measures the impact of migraine across three essential aspects of a patient’s health-related quality of life over the past 4 weeks: role function-restrictive (RR), role function-preventive (RP), and emotional function (EF). Raw dimension scores are computed as a sum of item response and rescaled from a 0 to 100 scale. The higher the score better is the quality of life [38].

**Fig 1:**
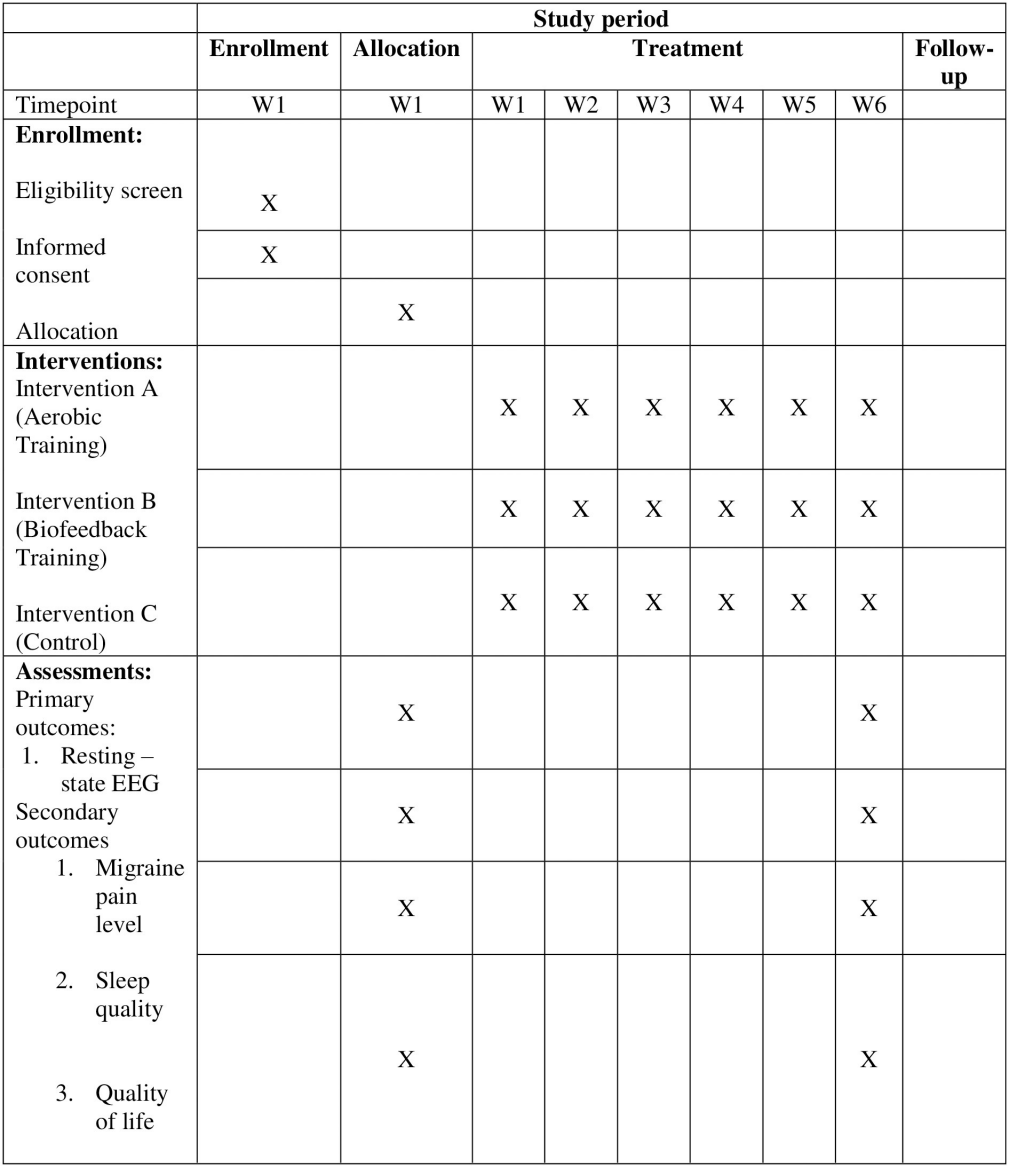
SPIRIT schedule of enrollment, interventions, and assessments.

**Fig 2:**
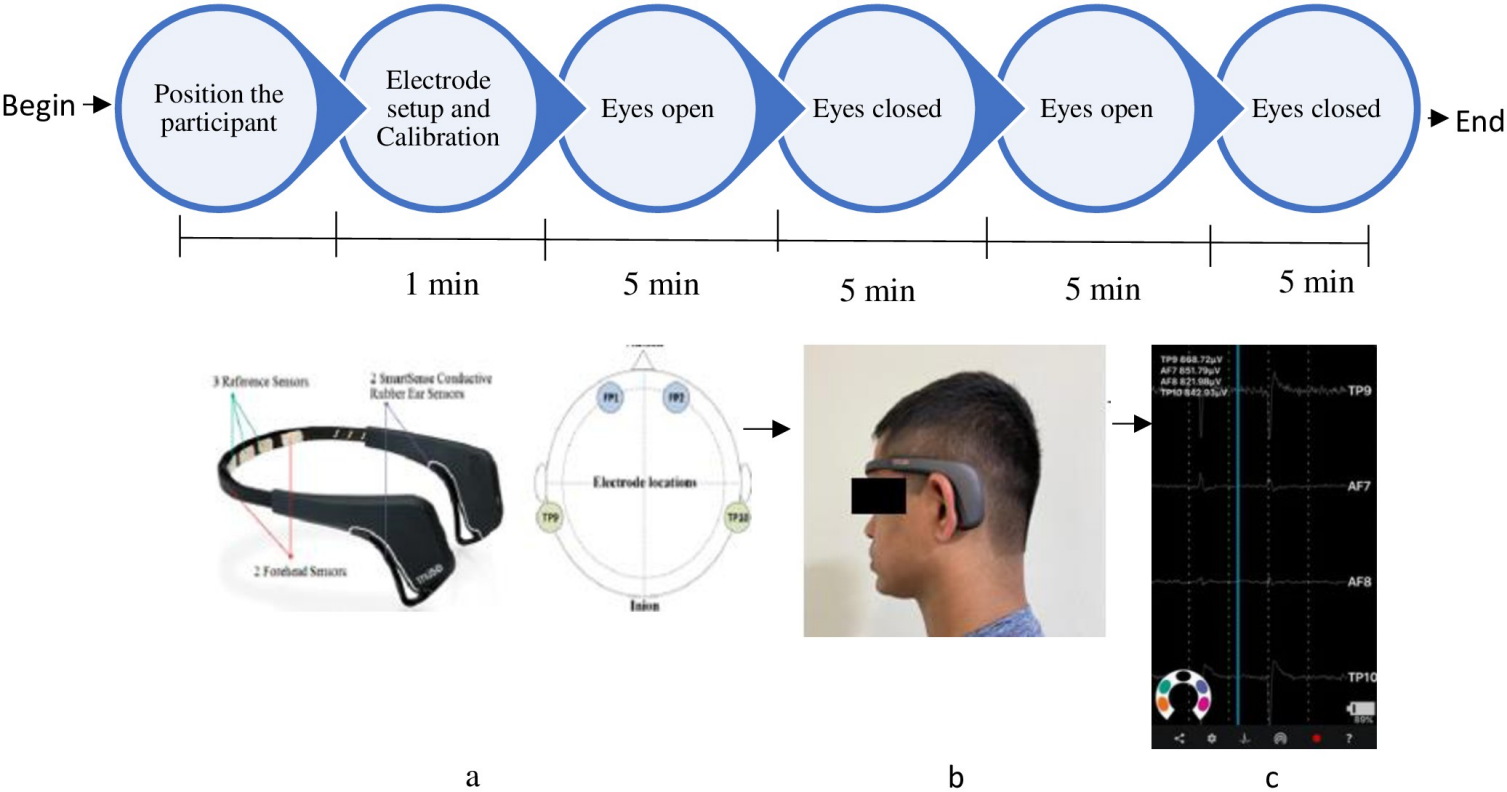
Protocol of resting-state EEG recording. a- MUSE 2 electrodes location, b- MUSE 2 setup, c- EEG recording from Mind Monitor App.

Recording of resting-state EEG will be performed using MUSE 2 a portable EEG recording device. The headset has four dry sensors (two mastoid and two forehead sensors). It fits over the ears and extends at an angle over the middle of the forehead when properly fitted with 3 reference electrodes. Once the headband is fitted, as shown in Fig 2b, the mind monitor app will be used for data acquisition. There will be horse to represent the sensors on the Muse and its connection. Solid ovals indicate a good connection, outlines indicate a poor connection and an empty space indicates no connection. The data acquisition space will be 100–200 cm from the computer/ tablet monitor displaying the stimulus presentation sequence. In the mind monitor application, the presentation of brain wave sequence can be in absolute, raw, discrete, accelerometer, and spectrogram views. In this study, raw EEG presentation values will be used for stimulus presentation sequence. So, each sensor’s raw data will be recorded in microvolts (μV). The sensors will be TP9 (left ear), AF7 (left forehead), AF8 (right forehead), TP10 (right ear), AUXR (right auxillay). The participant will be instructed not to clench teeth and blink eyes often since it can alter the activity recorded. The resting-state EEG will be recorded for a total of 20 minutes alternating between eyes open and eyes closed condition [39–41]. Details of the SPIRIT enrollment and the experimental protocol are as illustrated in Figs 1 and 2.

### Procedure and stages

**Fig 3:**
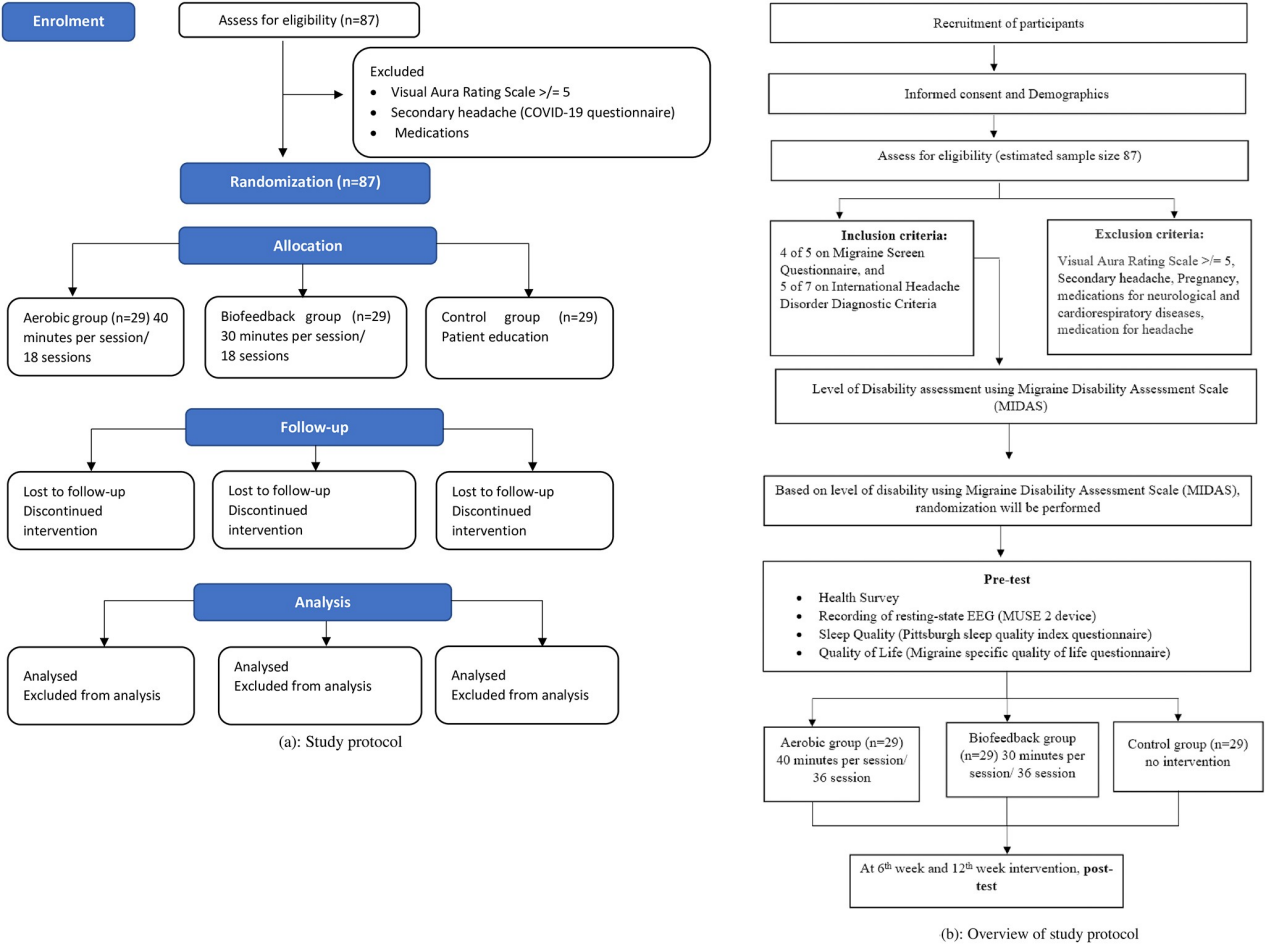
(a): Study protocol (b): OverView of study protocol.

Fig 3(a) and 3(b) summarizes the consort diagram and overview of the procedure to explain the study planned.

Volunteer of undergraduate and postgraduate students will be recruited as participants, and a questionnaire will be distributed to confirm the migraine symptoms among students in UTAR Sungai Long campus using Migraine Screen and ICHD 3 Questionnaires additionally, these participants will be assessed for exclusion criteria. Once confirmed with migraine symptoms, the level of disability using the Migraine Disability Assessment Scale questionnaire (MIDAS) will be assessed. The subjects in MIDAS I-IV will be randomly allocated to Group I: Aerobic training, Group II: Biofeedback training, Group III: placebo group.

The physiotherapy intervention phase will be initiated. The pre-test outcome measures will be assessed using 4-point pain intensity scale, the Pittsburgh Sleep Quality Index, Migraine Specific Quality of Life questionnaires, and recording of resting-state EEG using the MUSE 2 instrument. On the first day of the study, participants’ migraine phases will be designated as inter-ictal, pre-ictal, ictal, or post-ictal based on the subjective questions. The ictal phase will be coded when the participant suffered a migraine attack on the day session. Pre-ictal and post-ictal phases will be coded when the migraine is within 36 h before or after an ictal phase. The inter-ictal phase will be coded in a pain-free period between the pre-ictal and post-ictal phases. During this intervention phase, the subjects in all groups will be requested to maintain a headache diary based on the national headache foundation [42]. The headache diary consists of date of migraine symptom, start time and end time of migraine, intensity (1–10 most severe being 10), preceding symptoms, medication (and dosage), relief of symptoms (complete/ moderate/ none), any other related activities/ exercises, supplements take, and other symptoms occurred (chest pain, shortness of breath, leg cramps/ fatigues). The outcome measures will be measured at baseline and at the end of the intervention at the 6^th^ week.

After 6 weeks of intervention, recording resting-state EEG, migraine pain level, sleep quality using Pittsburgh sleep quality index and QoL using Migraine specific quality of life will be assessed and subjected to analyses.

### Protocol

#### Aerobic training

**Fig 4:**
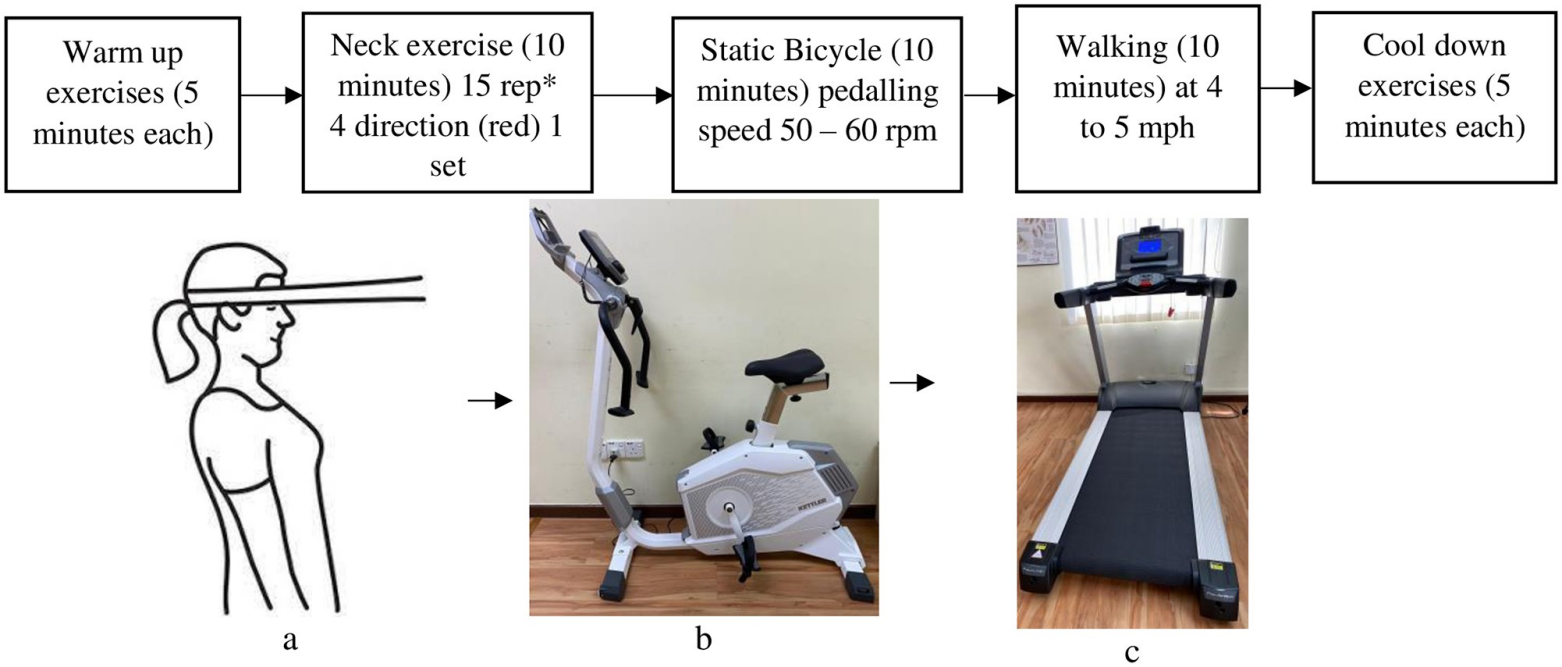
Protocol of aerobic training. a- Neck exercise with resistance band, b- Static bicycle, c- Treadmill.

Participants in the aerobic group will undergo training that includes neck exercise, static bicycle, and walking. The participants start the session with a warm-up for 5 minutes followed by 30 minutes of aerobic exercise and end with 5 minutes of cool-down exercise. 40 minutes/ session, 3 times per week for 6 weeks. Fig 4 summarizes the exercise protocol involved in the aerobic training group.

**Fig 5:**
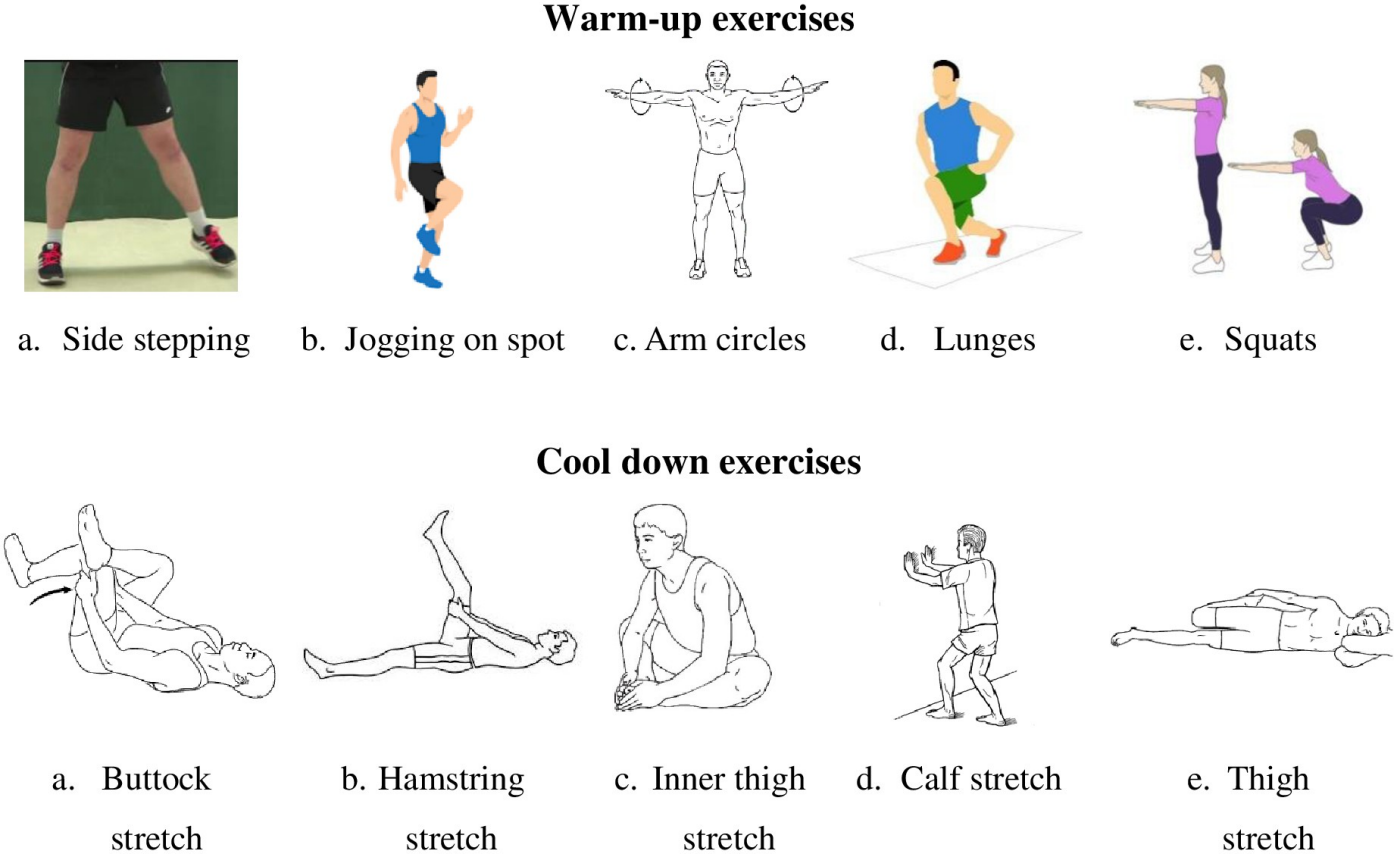
Warm up and cool down exercise.

The warm-up exercise will include light aerobic activity and some dynamic stretching movements. The exercises included will be fast-paced side steeping, jogging on the spot, arm swings, lunges, and squats, exercise to be performed for 10 repetition, each* 3 sets/ 1 minute each [43]. Similarly, the cool-down exercises will include buttock stretch, hamstring stretch, inner thigh stretch, calf stretch, and thigh stretch. Each stretch to be performed for 5 repetitions with a hold time of 15 seconds for each [44]. Fig 5 summarizes the warm-up and cool-down exercises.

During the exercise, the subjective measure of how hard the participant feels during the activity is measured using Borg’s Rating of Perceived Exertion (RPE) [45,46].

Similarly, during aerobic training, the exercise intensity will correlate with how hard the activity feels. The objective measurement of exercise intensity using heart rate calculation method will be used in this study. To calculate the heart rate, the Karvonen method, otherwise called as heart rate reserve (HRR) formula, takes the resting heart rate into consideration by introducing the difference between maximum heart rate and resting heart rate. Using the Karvonen principle, the target heart rate will be calculated using the formula: [(max HR − resting HR) × %Intensity] + resting HR. The maximum heart rate (max HR) will be calculated using the formula: 220-age. The resting heart rate (resting HR) will be calculated by measuring pulse at rest. For moderate-intensity physical activity, the target heart rate should be between 64% and 76% of the maximum heart rate [47].

An elastic band secured around the head will apply local pressure over the area. Red color bands will be used since the target area is muscle around the chin or shoulder. This strengthening exercise will use a dynamic isometric thera-band exercise that will be performed in a sitting position with a single series/ set of 15 repetitions in forward, obliquely toward the right and left, and backward.

Kettler Computeranleitung Advanced display will be used to train static cycling. Upon starting the machine, a new user item will be created, and the desired heart rate will be entered. During training, the participant grips the detector and starts cycling, Borg’s RPE will be maintained at 11–14. The central graphic in the display shows whether the participant is within range or whether above or below the target range. The average exercise bike pedaling speed will be 50 to 60 rpm.

ProAction BH Treadmill G6700 instrument will be used for aerobic training. Following the target heart rate calculation, the participant will start to walk at a comfortable pace by griping the detector, and Borg’s Rating of Perceived Exertion (RPE) measures the physical activity intensity level will be maintained at 11–14, and the speed of walking will be at 4 mph and depending on RPE response the speed will be either be maintained or increased to 5 mph.

#### Biofeedback training

**Fig 6:**
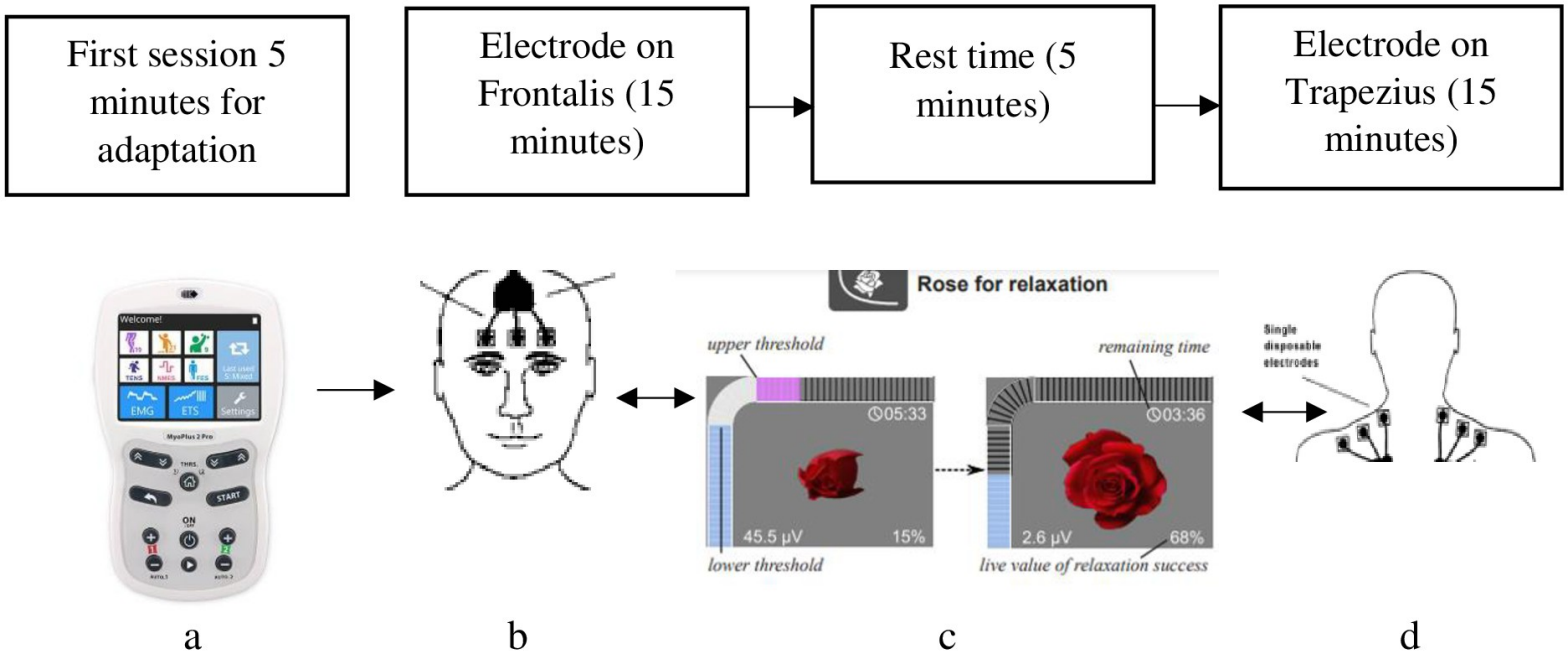
Protocol of biofeedback training. a- biofeedback training device, b- frontalis electrdode placement, c- training device display/feedback, d- trapezius electrode placement.

Participants in this group will undergo an electromyography (EMG) biofeedback training for the trapezius and frontalis using a rose for relaxation 3 times per week for 6 weeks. Each session will be 30 minutes, with a 5-minute resting period between each muscle session. Fig 6 summarizes the biofeedback training protocol.

Neurotrac myoplus 4 pro instrument will be used for EMG biofeedback training. The rose game for relaxation provides an EMG biofeedback when the participant’s rose opens when relaxation is detected, and the aim is to relax completely and stay relaxed. 4 microvolt is the default value which can still be lowered one’s relaxation ability is achieved. During each training session, the electrodes will be placed on Frontalis and Trapezius muscle. The electrodes are connected to this machine that gives feedback to the participant via a visual signal, allowing them to decrease or increase muscle activity. During the training sessions, the patient will be made to sit in a comfortable chair with a straight back, feet flat on the floor (forming 90 –degrees with knees and with arm-rest and instructed to maintain erect sitting throughout [48].

### Placebo group

The placebo group will receive the patient education sheet with basic information about migraine in terms of symptoms, triggers, and prevention tips.

### Compliance during the experimental study

The participants need to complete 18 sessions within 6 weeks duration (3 times a week or alternative days)

#### Termination criteria

The termination criteria are based on ACSM guidelines for exercise testing/ prescription [49] and migraine trust [50]:

Absolute termination: Onset of moderate to severe angina [51], signs of poor perfusion: pale appearance to the skin, bluish discoloration, excessive cold and clammy skin, severe or unusual shortness of breath (modified Borg’s scale), ataxia (failure of muscle coordination), vertigo (an illusion of dizzying movement), visual or gait problems and confusion Migraine induced headache Relative termination: Any chest pain that is increasing shortness of breath (modified Borg’s scale), leg cramps/ fatigue, patients’ request.

#### Safety consideration

The participants will be encouraged to: Wear comfortable clothing and well-padded shoes that can protect the heels and arches of their feet. Perform warm-up before doing aerobic exercises and cool-down after exercises to reduce the risk of strains and sprains. If there is dizziness, shortness of breath, chest pain, nausea, vomiting, and joint pain during excise, stop the activity. The temperature in the aerobic area is to be maintained at a moderate level, with good ventilation. Schedule regular days off from exercise and rest when tired.

### Statistical methods

#### Analysis of EEG signals

Amplitude and frequency, frequency band ratio, power spectrum density, and coherence will be analysed for resting-state EEG recording. The recorded data from the MUSE 2 portable EEG device can be further analysed in MATLAB. The data obtained will be imported to MATLAB using EEGLAB function plugins (muse monitor app) CSV file. Once imported, the pre-processing steps will be done to run Independent Component Analysis (ICA). The sampling frequency will be set at 256Hz. To remove linear trends, high-pass filtering at 1 Hz will be done to obtain good quality Independent Component Analysis (ICA) decompositions. Average referencing for source localization will be done since a non-scalp reference is used. Artifact rejection and ICA will be used to remove artifacts such as muscle, eye blinks, or eye movements without removing the affected data portions.

After extracting epochs and plotting data, the EEG amplitude, frequency, frequency band ratio, power spectrum density, and coherence will be analysed. EEG will be decomposed into the five EEG sub-bands of frequency and amplitude: delta (0.5–3 Hz/ 5–250 microvolt), theta (4–7 Hz/ 20–100 microvolt), alpha (8–12 Hz/ 5–120 microvolt), beta (13–30 Hz/ 5–50 microvolt) and gamma (31–40 Hz/ -10 microvolt). MATLAB will design a filter with the ’butter’ command for frequency band ratio, setting the cut-off frequencies according to alpha or theta band or appropriate bands and then using it on data with the ’filter’ command.

The power spectral density of EEG will be estimated using AR Burg method. The AR method is based on modeling the data sequence x(n) as the output of a causal and discrete filter whose input is white noise, which is expressed as the follows:

$$x\left(n\right)=-\overset{p}{\underset{k=1}{\sum }}a\left(k\right)\cdot x\left(n-k\right)+\omega \left(n\right)$$

where a(k) is the AR coefficient, x(n) is the white noise of variance equal to r2, ω(n) is window function and p is the order of the AR model.

Next, the coherence estimation will be performed. Coherence represents the normalized covariance of two time series in the frequency domain. Mathematically, the coherence function C_xy_(f) at a frequency f for signal x and y is obtained by the normalization of the cross-spectral spectrum as follows:

$$C_{xy}\left(f\right)=\frac{\left|\langle P_{xy}\left(f\right)\rangle \right|}{\sqrt{P_{xx}\left(f\right)}*\sqrt{P_{yy}\left(f\right)}}$$

### Statistical analyses

The planned statistical analysis for each phase: Data collected will be computed and analyzed using IBM Statistical Package for Social Science (SPSS) version 22.0 to produce the study results. Descriptive analyses using the mean and the standard deviation will be used to examine the data obtained. Descriptive statistics will be used to analyse the demographic data of every subject at their baseline. A box plot graph will be used to portray the distribution of data for example age, gender, year of study, area of study, and physical activity level.

The distribution of data will be analysed using normality of distribution. Mixed design analysis and intention-to-treat analysis will be used to assess the efficacy of aerobic training.

## Discussion

There is no specific test to diagnose migraine. For an accurate diagnosis, a general physician must identify the pattern and associated symptoms. Migraines can be unpredictable, sometimes occurring without symptoms. If underdiagnosed or over-looked, it encompasses a serious of long-term effects. "Studies show a dysfunctional learning process in the brain in migraine and in other pain conditions," Brennan says: "The brain learns to produce and perpetuate pain." In other words, migraine can teach the brain that pain is normal, so the brain changes to help pain happen more often [52]. The long-term comorbidities include cardiovascular disease, stroke, white matter lesions, hypertension, gene polymorphisms, cardiac disease, psychiatric disease, depression, diffuse anxiety disorder, panic disorder, and bipolar disorder [53], obesity. Thus, a non-pharmacological treatment becomes more evident to avoid polypharmacy or drug interactions [54].

Hence with appropriate intervention, the symptoms can be prevented from worsening. Evidence suggests the importance of psychological [5], behavioural [55], and surgical intervention [14]. But there is an unmet need for evidence-based non-pharmacological approaches to complement pharmacotherapy in migraine prevention [54]. On the other hand, some migraineurs report exercise as a triggering factor for their attacks [56]. Maybe this is one of the reasons for lacking evidence. In general, exercise can be used for the management of several chronic pain conditions [57]. Moreover, an exercise intervention may be more suitable for people with migraine considering their tendency toward inactivity [54]. The empirical support for recommending a specific exercise program for prophylactic treatment is relatively limited [54]. Hence this is the first study also aiming to establish an aerobic protocol for patients with migraine symptoms. The intervention proposed in this study is simple and would be easy to implement in a clinical setup provided it if shown to be effective. The failure of more intensive interventions may be due to lack of compliance [58]. Thus, this study chose an intervention level to achieve a high compliance rate.

Although some studies developed exercise programs for untrained patients with migraine, the outcome was primarily in terms of exercise capacity rather than the primary characteristics and secondary resting-state brain wave analysis/ sleep quality changes, indicating the need for this study. Additionally, these studies assessed the impact of drugs [27] indicating the need to assess the non-pharmacological importance to migraine patients. One of the expectations in this study is that the participants would also prefer to modify their existing lifestyle and thus will have several health benefits.

## Supporting information

S1 Checklist SPIRIT 2013 checklist: Recommended items to address in a clinical trial protocol and related documents*. (DOC)

S1 File (DOCX)

S2 File (PDF)

S3 File (DOCX)
