# Supplementary material for: Preliminary efficacy of aerobic training among university students with migraine symptoms: Study protocol for a pilot randomized controlled trial
Source: PLoS One. 2023 Sep 25;18(9):e0291534. doi: 10.1371/journal.pone.0291534 (PMC10519594; doi:10.1371/journal.pone.0291534)
Supplement: S1 Checklist — (DOC) [file pone.0291534.s001.doc]

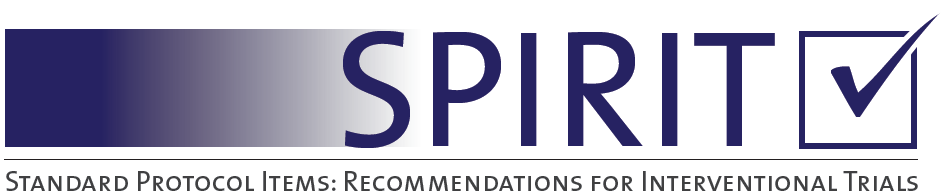


SPIRIT 2013 Checklist: Recommended items to address in a clinical trial protocol and related documents*

| Section/item | ItemNo | Description |
| --- | --- | --- |
| **Administrative information** | | |
| Title | 1 | **Descriptive title identifying the study design, population, interventions, and, if applicable, trial acronym**  Efficacy of Physiotherapy intervention among university students with migraine symptoms: study protocol for Randomized Control Trail |
| Trial registration | 2a | **Trial identifier and registry name. If not yet registered, name of intended registry**  The study is registered with ClinicalTrials.gov Protocol Registration and Results System  Identifier: NCT05741775 |
|  | 2b | **All items from the World Health Organization Trial Registration Data Set**  Nil |
| Protocol version | 3 | **Date and version identifier**  2022, version 1 |
| Funding | 4 | **Sources and types of financial, material, and other support**  This project is funded by UTAR RESEARCH FUND, project number: IPSR/RMC/UTARRF/2022-C2/K02 |
| Roles and responsibilities | 5a | **Names, affiliations, and roles of protocol contributors**   1. Kiruthika Selvakumar (primary investigator): Lecturer, Department of Physiotherapy, M. Kandiah Faculty of Medicine and Health Sciences, Universiti Tunku Abdul Rahman, Selangor, Malaysia. (Conceptualization, data curation, methodology, writing) 2. Tan Lee Fan (Supervisor): Assistant Professor, Department of Mechatronics and BioMedical Engineering, Lee Kong Chian Faculty of Engineering and Science, Universiti Tunku Abdul Rahman, Selangor, Malaysia   (Formal analysis, software, supervision, validation)   1. Foo Chai Nien (Co-supervisor): Assistant Professor, Department of Population Medicine, M. Kandiah Faculty of Medicine and Health Sciences, Universiti Tunku Abdul Rahman, Selangor, Malaysia   (Formal analysis, software, supervision, validation)   1. Mun Hou Kit (EEG/ MATLAB specialist): Assistant Professor, Department of Mechatronics and BioMedical Engineering, Lee Kong Chian Faculty of Engineering and Science, Universiti Tunku Abdul Rahman, Selangor, Malaysia   (Formal analysis, software, supervision, validation) |
|  | 5b | **Name and contact information for the trial sponsor**  Institute of Postgraduate Studies and Research, Universiti Tunku Abdul Rahman (UTAR) |
|  | 5c | **Role of study sponsor and funders, if any, in study design; collection, management, analysis, and interpretation of data; writing of the report; and the decision to submit the report for publication, including whether they will have ultimate authority over any of these activities**  Sponsor for the following:  Research material and supplies (V26000) (MUSE 2 portable EEG device)  Special service (V29000) (Refreshments of participants) |
|  | 5d | **Composition, roles, and responsibilities of the coordinating centre, steering committee, endpoint adjudication committee, data management team, and other individuals or groups overseeing the trial, if applicable (see Item 21a for data monitoring committee)**  The primary investigator and research assistant will help in liaising with participants for each session and thereby maintaining the sessions. |
| Introduction |  |  |
| Background and rationale | 6a | **Description of research question and justification for undertaking the trial, including summary of relevant studies (published and unpublished) examining benefits and harms for each intervention**  Habitual aerobic exercise has a major advantage of preventing or reducing symptoms of several chronic diseases and medical conditions. Aerobic exercises have already proven to reduce frequency, duration, severity or associated disability in migraine. The physiology is that when one exercises, the body releases endorphins, which are the body's natural painkillers and natural anti-depressants chemicals called enkephalins. According to Centre of Disease Control and Prevention (CDC), adult should exercise 150 minutes of moderate intensity aerobic exercise and 2 or more days a week of muscle strengthening each week for relief of migraine or primary headaches. For this reason, several studies encourage a multidisciplinary and tailored treatment approach for these patients. But the long term effects of these interventions especially on quality of life are less commonly studied. Hence the study aim to assess the efficacy of physiotherapy intervention especially aerobic exercise among university students with migraine symptoms. |
|  | 6b | **Explanation for choice of comparators**  A systematic analysis published in 2019, concluded that none of the included studies in this narrative review met valid criteria of good clinical practice that assess the impact of aerobic exercise on migraine. Indicating the need for further analysis.  According to national institute of health, for biofeedback training, it is believed to have a beneficial effect on autonomous nervous system activity and render individuals resilient to stressors that may trigger a migraine. |
| Objectives | 7 | **Specific objectives or hypotheses**  The goal of this randomized control trial is to analyse the effectiveness of Physiotherapy intervention among university students with migraine symptoms. The main objective is:   - To determine the effect of aerobic exercise on the resting-state brainwaves among university students in UTAR with migraine symptoms compared with biofeedback and control exercise. - To analyse the influence of aerobic exercise on the sleep quality and quality of life among the cohort compared with biofeedback and control exercise.   Although the Migraine Research Foundation listed three main types of non-drug treatments for migraine are lifestyle advice, therapies, and exercises. Some common aerobic exercises such as walking, jogging, a behavioral weight loss program, cycling, and a combination of cross-training, walking, jogging, and cycling are suggested to be beneficial to the migraine patients but there remains no specific protocol established till now. Hence the other main objective of this is to establish a aerobic exercise protocol for patients with migraine symptoms. |
| Trial design | 8 | **Description of trial design including type of trial (eg, parallel group, crossover, factorial, single group), allocation ratio, and framework (eg, superiority, equivalence, noninferiority, exploratory)**  Design: Pre-and post-test RCT design  Study Type: Interventional (Clinical Trial)  Allocation: Randomized  Intervention Model: Parallel Assignment  Estimated Enrollment :87 participants (Equivalence) |

| Methods: Participants, interventions, and outcomes | | |
| --- | --- | --- |
| Study setting | 9 | **Description of study settings (eg, community clinic, academic hospital) and list of countries where data will be collected. Reference to where list of study sites can be obtained**  This study will be carried out at Physiotherapy Center, Universiti Tunku Abdul Rahman, Sungai Long Campus. The data will be collected in Malaysia only. |
| Eligibility criteria | 10 | **Inclusion and exclusion criteria for participants. If applicable, eligibility criteria for study centres and individuals who will perform the interventions (eg, surgeons, psychotherapists)**  Inclusion Criteria:  Undergraduate, and postgraduate students from Universiti Tunku Abdul Rahman  4 of 5 on the Migraine Screen Questionnaire  Exclusion Criteria:  Score of more than or equal to 5 on the visual aura rating scale,  Diagnosed to have a secondary headache (headache attributed to the causative disorder example: infection, trauma, injury to head/ or neck)  Pregnancy,  Took medication for neurological conditions like stroke, multiple sclerosis and took medications for cardiorespiratory conditions like asthma,  Took medications for headache and  Unwilling to participate will be excluded |
| Interventions | 11a | **Interventions for each group with sufficient detail to allow replication, including how and when they will be administered**  Experimental: Aerobic training  Participants in the aerobic group will undergo a training that includes walking, static bicycle, and neck exercise. The participants start the session with a warm-up for 5 minutes followed by 30 minutes of aerobic exercise and end with 5 minutes of cool-down exercise. 40 minutes/ session, 3 times per week for 6 weeks.  Experimental: Biofeedback training  Participants in this group will undergo an electromyography (EMG) biofeedback training for trapezius and frontalis using rose for relaxation 3 times per week for 6 weeks. Each session will be for 30 minutes with a 5-minute resting period between each muscle session.  No Intervention: Waitlist Control group  The control group will receive the patient education sheet with the basic information about migraine in terms of symptoms, triggers, and prevention tips. This group is also called as "waitlist control group" who will receive intervention after the active treatment group. |
|  | 11b | **Criteria for discontinuing or modifying allocated interventions for a given trial participant (eg, drug dose change in response to harms, participant request, or improving/worsening disease)**  The termination criteria are based on ACSM guidelines for exercise testing/ prescription and migraine trust:  Absolute termination: Onset of moderate to severe angina (46), Signs of poor perfusion: pale appearance to the skin, bluish discoloration, excessive cold and clammy skin, severe or unusual shortness of breath (modified Borg’s scale), ataxia (failure of muscle coordination), vertigo (an illusion of dizzying movement), visual or gait problems and confusion Migraine induced headache.  Relative termination: Any chest pain that is increasing shortness of breath (modified Borg’s scale), Leg cramps/ fatigue, patients’ request |
|  | 11c | **Strategies to improve adherence to intervention protocols, and any procedures for monitoring adherence (eg, drug tablet return, laboratory tests)**  Quality controller will be appointed to check the adherence to the intervention protocol. |
|  | 11d | **Relevant concomitant care and interventions that are permitted or prohibited during the trial**  Nil |
| Outcomes | 12 | **Primary, secondary, and other outcomes, including the specific measurement variable (eg, systolic blood pressure), analysis metric (eg, change from baseline, final value, time to event), method of aggregation (eg, median, proportion), and time point for each outcome. Explanation of the clinical relevance of chosen efficacy and harm outcomes is strongly recommended**  **Primary Outcome Measures:** Resting-state EEG [ Time Frame: 20 minutes ]  Recording of resting-state EEG will be performed using MUSE 2 a portable EEG recording device. Once the headband is fitted, the mind monitor app will be used for data acquisition. The data obtained will be imported to MATLAB using EEGLAB function plugins (muse monitor app) CSV file. Once imported the pre-processing steps will be done to run Independent Component Analysis (ICA). Amplitude and frequency, frequency band ratio, power spectrum density and coherence will be the primary measurement analyzed for the resting-state EEG recording,  **Secondary Outcome Measures:**   1. Pittsburgh Sleep index [ Time Frame: 10 minutes ]   Pittsburgh Sleep index is a self-rated questionnaire that assesses sleep quality and disturbances over a 1-month time interval. 19 individual items generate seven component scores: subjective sleep quality, sleep latency, sleep duration, habitual sleep efficiency, sleep disturbances, use of sleeping medication, and daytime dysfunction. The sum of scores for these seven components yields one global score. The sleep component scores are summed to yield a total score ranging from 0 to 21 with the higher total score (referred to as global score) indicating worse sleep quality.   1. Migraine Specific Quality of life [ Time Frame: 10 minutes ]   Migraine Specific Quality of life is a 14-item instrument that measures the impact of migraine across three essential aspects of a patient's health-related quality of life over the past 4 weeks: role function-restrictive (RR), role function-preventive (RP), and emotional function (EF). Raw dimension scores are computed as a sum of item response and rescaled from a 0 to 100 scale. The higher the score better is the quality of life.   1. Health survey questionnaire [ Time Frame: 5 minutes ]   Characteristics like frequency, severity and duration of migraine. Level of pain scored on a four-point numerical rating scale (0-3) equivalent to no, mild, moderate, and severe pain: 0 no pain. 1 mild pain, does not interfere with usual activities 2 moderate pain, inhibits but does not wholly prevent usual activities 3 severe pain, prevents all activities. The other components are expressed as either decreased/ increased/ remains the same/ unable to recall. |
| Participant timeline | 13 | **Time schedule of enrolment, interventions (including any run-ins and washouts), assessments, and visits for participants. A schematic diagram is highly recommended (see Figure)**  **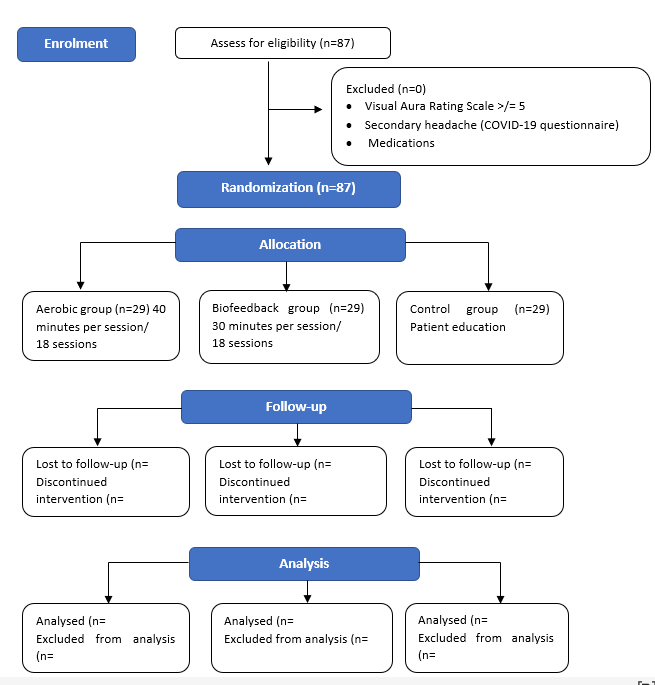**  Actual Study Start Date: September 18, 2022  Estimated Primary Completion Date: September 2023  Estimated Study Completion Date: December 2024  No washouts, currently in Early Phase 1 enrolment and intervention |
| Sample size | 14 | **Estimated number of participants needed to achieve study objectives and how it was determined, including clinical and statistical assumptions supporting any sample size calculations**  Sample size is estimated based on G*power statistical power analysis program software version 3.1.9.7. Using F test and statistical method as ANOVA: fixed effects, omnibus, one way with effect size of 0.49, alpha error probability 0.05, power (1-beta error probability) 0.95 and number of groups being 3, the total size estimated is 69. Then, further adjustment of sample size to accommodate 20% dropout rate (26) gives the final estimated value to 87. |
| Recruitment | 15 | **Strategies for achieving adequate participant enrolment to reach target sample size**  Registration form will be circulated via email, WhatsApp to achieve the target sample size. |
| **Methods: Assignment of interventions (for controlled trials)** | | |
| Allocation: |  |  |
| Sequence generation | 16a | **Method of generating the allocation sequence (eg, computer-generated random numbers), and list of any factors for stratification. To reduce predictability of a random sequence, details of any planned restriction (eg, blocking) should be provided in a separate document that is unavailable to those who enrol participants or assign interventions**  The subjects will be given a random number in an excel sheet and ranked. Following ranking, the ranked participant will be divided by size and group accordingly. |
| Allocation concealment mechanism | 16b | **Mechanism of implementing the allocation sequence (eg, central telephone; sequentially numbered, opaque, sealed envelopes), describing any steps to conceal the sequence until interventions are assigned**  Blinded participants, excel random number |
| Implementation | 16c | **Who will generate the allocation sequence, who will enrol participants, and who will assign participants to interventions**  Primary investigator |
| Blinding (masking) | 17a | **Who will be blinded after assignment to interventions (eg, trial participants, care providers, outcome assessors, data analysts), and how**  The participant will not know whether he or she belongs to experimental group. Only the primary researcher performing the study will know if the participants are into aerobic training, biofeedback training or control group. Single blinding will be used in this study because the results produced are less likely to be biased. |
|  | 17b | **If blinded, circumstances under which unblinding is permissible, and procedure for revealing a participant’s allocated intervention during the trial**  Unblinding is not permissible, since the psychology can also affect the outcomes. |
| **Methods: Data collection, management, and analysis** | | |
| Data collection methods | 18a | **Plans for assessment and collection of outcome, baseline, and other trial data, including any related processes to promote data quality (eg, duplicate measurements, training of assessors) and a description of study instruments (eg, questionnaires, laboratory tests) along with their reliability and validity, if known. Reference to where data collection forms can be found, if not in the protocol**  Each participant will be assessed for primary and secondary outcomes. Sessions for the experimental group will be supported by training an assessor (assistant). The data recorded will be accessible by primary investigator, supervisor and co-supervisor. |
|  | 18b | **Plans to promote participant retention and complete follow-up, including list of any outcome data to be collected for participants who discontinue or deviate from intervention protocols**  The participants need to complete 18 sessions within 6 weeks duration (3 times a week or alternative days). At least 80% of session need to be completed. If unable to complete, the data received will be considered as incomplete follow up and will not be proceeded for further analysis. |
| Data management | 19 | **Plans for data entry, coding, security, and storage, including any related processes to promote data quality (eg, double data entry; range checks for data values). Reference to where details of data management procedures can be found, if not in the protocol**  Personal laptop and official student ID drive. |
| Statistical methods | 20a | **Statistical methods for analysing primary and secondary outcomes. Reference to where other details of the statistical analysis plan can be found, if not in the protocol**  Statistical analysis method is enclosed in the protocol. |
|  | 20b | **Methods for any additional analyses (eg, subgroup and adjusted analyses)**  Nil |
|  | 20c | **Definition of analysis population relating to protocol non-adherence (eg, as randomised analysis), and any statistical methods to handle missing data (eg, multiple imputation)**  Any missing data, non-adherence to the protocol will be considered as missing data and will be excluded from further analysis. |
| **Methods: Monitoring** | | |
| Data monitoring | 21a | **Composition of data monitoring committee (DMC); summary of its role and reporting structure; statement of whether it is independent from the sponsor and competing interests; and reference to where further details about its charter can be found, if not in the protocol. Alternatively, an explanation of why a DMC is not needed**  Quality controller will help in maintaining the planned interventions. The quality controller checklist will consist of general information relating to protocol, procedure, consistency, guidelines. Randomization, data collection, treatment of subjects, assessment of efficacy, assessment of safety, data handling and record keeping, ethics and finance and insurance will be covered. The quality controller will also provide additional comments for each section and depending on individual participants. |
|  | 21b | **Description of any interim analyses and stopping guidelines, including who will have access to these interim results and make the final decision to terminate the trial**  The intervention will be terminated when participant have these following absolute contra indication. Onset of moderate to severe angina, signs of poor perfusion: pale appearance to the skin, bluish discoloration, excessive cold and clammy skin, severe or unusual shortness of breath (modified Borg’s scale), ataxia (failure of muscle coordination), vertigo (an illusion of dizzying movement), visual or gait problems and confusion migraine induced headache. |
| Harms | 22 | **Plans for collecting, assessing, reporting, and managing solicited and spontaneously reported adverse events and other unintended effects of trial interventions or trial conduct**  Upon recruitment and voluntary consent, each participant will be given an headache diary to monitor the symptoms for next 6 weeks. The headache diary consists of date of migraine symptom, start time and end time of migraine, intensity (1 – 10 most severe being 10), preceding symptoms, medication (and dosage), relief of symptoms (complete/ moderate/ none), any other related activities/ exercises, supplements take, and other symptoms occurred (cheat pain, shortness of breath, leg cramps/ fatigues). |
| Auditing | 23 | **Frequency and procedures for auditing trial conduct, if any, and whether the process will be independent from investigators and the sponsor**  Yes, the quality control will check the protocol, sessions, feasibility and provide suggestions. |
| Ethics and dissemination | | |
| Research ethics approval | 24 | **Plans for seeking research ethics committee/institutional review board (REC/IRB) approval**  The study is approved by UTAR Scientific and Ethical Review Committee (U/SERC/188/2022) |
| Protocol amendments | 25 | **Plans for communicating important protocol modifications (eg, changes to eligibility criteria, outcomes, analyses) to relevant parties (eg, investigators, REC/IRBs, trial participants, trial registries, journals, regulators)**  To the same UTAR Scientific and Ethical Review Committee, the ethical to be renewed every year and any change in the protocol will be considered as new submission and not renewal of ethical. |
| Consent or assent | 26a | **Who will obtain informed consent or assent from potential trial participants or authorised surrogates, and how (see Item 32)**  Primary investigator |
|  | 26b | **Additional consent provisions for collection and use of participant data and biological specimens in ancillary studies, if applicable**  Not applicable |
| Confidentiality | 27 | **How personal information about potential and enrolled participants will be collected, shared, and maintained in order to protect confidentiality before, during, and after the trial**  Participants data will be collected via hard copy (consent form and data protection), google link for pre and post-test questionnaires and a mind monitor app (csv) file for resting state brain wave data analysis. The data collected will be available only in personal laptop and official student ID drive. |
| Declaration of interests | 28 | **Financial and other competing interests for principal investigators for the overall trial and each study site**  Nil |
| Access to data | 29 | **Statement of who will have access to the final trial dataset, and disclosure of contractual agreements that limit such access for investigators**  Primary investigator |
| Ancillary and post-trial care | 30 | **Provisions, if any, for ancillary and post-trial care, and for compensation to those who suffer harm from trial participation**  Fatigue (tiredness) following exercises, hence no post-trail care is needed in this study. |
| Dissemination policy | 31a | **Plans for investigators and sponsor to communicate trial results to participants, healthcare professionals, the public, and other relevant groups (eg, via publication, reporting in results databases, or other data sharing arrangements), including any publication restrictions**  Via publication. Individual reports/ data sheet will not be disclosed in any research article, instead the grand average of the outcome swill be presented. |
|  | 31b | **Authorship eligibility guidelines and any intended use of professional writers**  No |
|  | 31c | **Plans, if any, for granting public access to the full protocol, participant-level dataset, and statistical code**  No |
| Appendices |  |  |
| Informed consent materials | 32 | **Model consent form and other related documentation given to participants and authorised surrogates**  The consent from will include the title of the research project, purpose of the study, procedure, risk and discomfort, benefits, payment, alternatives, contact person and consent. |
| Biological specimens | 33 | **Plans for collection, laboratory evaluation, and storage of biological specimens for genetic or molecular analysis in the current trial and for future use in ancillary studies, if applicable**  Not applicable |

*It is strongly recommended that this checklist be read in conjunction with the SPIRIT 2013 Explanation & Elaboration for important clarification on the items. Amendments to the protocol should be tracked and dated. The SPIRIT checklist is copyrighted by the SPIRIT Group under the Creative Commons “[Attribution-NonCommercial-NoDerivs 3.0 Unported](http://www.creativecommons.org/licenses/by-nc-nd/3.0/)” license.
