# Supplementary material for: Preliminary efficacy of aerobic training among university students with migraine symptoms: Study protocol for a pilot randomized controlled trial
Source: PLoS One. 2023 Sep 25;18(9):e0291534. doi: 10.1371/journal.pone.0291534 (PMC10519594; doi:10.1371/journal.pone.0291534)
Supplement: S1 File — (DOCX) [file pone.0291534.s002.docx]

# Efficacy of physiotherapy intervention among university students with migraine symptoms

1. **Introduction**

Migraine is a prevalent disorder, affecting 15.1% of the world’s population. According to the International Classification of Headache Disorders, 3^rd^ edition, migraine is classified into migraine without aura and migraine with aura. Migraine is characterized by multiple phases: premonitory, aura, headache, postdrome, and interictal (Dodick, 2018). According to DaSilva during a migraine attack, the patients’ dopamine levels cause the sensory structures to be more sensitive, so that normally painless sensory signals from the skin, muscle, and blood vessels, and other structures would become more painful (Bailey, 2017). The excitable brain cells produce a continuous output of small electrical signals, often referred to as brain waves. Brain waves are oscillating electrical voltages in the brain measuring just a few millionths of a volt. The recording of these signals called an electroencephalogram (EEG), is the summation of all postsynaptic potential of neurons in the cerebral cortex (Snyder, 1990). The frequently used method to classify EEG waveforms is by frequency. The most studied waveform is Delta (0.5 to 4Hz) physiologically seen in deep sleep and is prominent in the frontocentral head regions. Theta (4 to 7Hz) brought on by drowsiness as well as early stages of sleep most prominent in the fronto-central head regions. Beta (13 to 30Hz) rhythm is the most frequently seen in normal adults and children. It is most prominent in the frontal and central head regions. Alpha (8 to 12Hz) the posterior dominant rhythm is characteristically present in normal awake EEG recordings in the occipital head region (Nayak & Anilkumar, 2020). Alpha brainwaves are slower, and higher in amplitude. Always the brain waves shift from one to another depending on the state of consciousness and alert. If the brain is not producing alpha waves, it can be concluded that participants are not in a relaxed, meditative state of mind.

Migraine is a leading cause of disability worldwide; approximately half of those affected have such severe attacks that they cannot function normally in routine daily activities (Brandes, 2009). According to the American migraine foundation, people with migraine are 2 to 8 times more likely to experience sleep disorders. The most common sleep disorders are insomnia, sleep apnoea, and teeth grinding. Disturbed sleep is a particularly common problem among migraineurs affecting 30% to 50% of migraine patients. Although there is no mortality related to primary headaches, the experience of pain of the patients with headaches, both disrupts the quality of life and puts a severe financial burden on the community (Taşkapilioǧlu & Necdet, 2013). The treatment for primary headaches is not common, it depends on severity, symptoms, and disability. Though pharmacological medications may be advised, they cannot be given for a longer duration due to their side effects. Thus, a non- pharmacological intervention is required which includes lifestyle advice, therapies, and exercises. The effectiveness of these therapies depends on the therapist, length of treatment, and commitment towards regular practice. Habitual aerobic exercise has the major advantage of preventing or reducing symptoms of several chronic diseases and medical conditions. Aerobic exercises have already proven to reduce frequency, duration, severity, or associated disability in migraine (Irby. et al., 2016). According to the Centre for Disease Control and Prevention, the adult should exercise 150 minutes of moderate-intensity aerobic exercise and 2 or more days a week of muscle-strengthening each week for relief of migraine or primary headaches. Hence, the purpose of this study is to investigate whether physiotherapy intervention influences brain activity, quality of life, and sleep disturbances among subjects with migraine-type primary headaches.

The novelty of the study as follows: The Phase 1 study will develop a new scale for measuring the impact of COVID-19 pandemic on existing migraine symptoms during diagnosed/ suspected periods of infection. The Phase 2 study will be the first interventional study with an active control to explore the effect of aerobic exercise on the quality of life, sleep pattern, and resting-state brain activity.

# Problem statement

To date, no interventional study has been carried out to analyse the effect of aerobic exercise on the quality of life, sleep pattern, and brain activity which are the important factors that determine the productivity of a person. Hence, the present study aims to explore the effect of aerobic exercise on these factors through an interventional setting.

# Research Question

- - - Does physiotherapy intervention have an impact on brainwaves, quality of life, sleep pattern among university students in UTAR with migraine symptoms?

# Research Objective

The major objective of this study is:

1. To determine the effect of aerobic exercise on the resting-state brainwaves among university students in UTAR with migraine symptoms.
2. To analyse the influence of aerobic exercise on the sleep quality and quality of life among the cohort.

# Hypothesis

Null hypothesis:

1. Aerobic exercise will show the same efficacy as control exercises indicated by improvement in the resting-state brainwaves among university students in UTAR with migraine symptoms.
2. Aerobic exercise will show the same efficacy as control exercises in improving the sleep quality and quality of life among the cohort.

Alternative Hypothesis:

- - 1. Aerobic exercise will be better than control exercises indicated by improvement in the resting- state brainwaves among university students in UTAR with migraine symptoms.
    2. Aerobic exercise will be better than control exercises in improving the sleep quality and quality of life among the cohort.

# Significance of the study

With the data obtained, further research can be the focus on developing and testing more effective and personalized rehabilitation or treatment approaches. The findings will lead to the understanding of neurobiological aspects of migraine pain and expand the horizon of understanding patients and not simply their headaches. Simultaneously, consequences of migraine symptoms on sleep, dietary habits, depression, general anxiety disorders, panic disorders can be analysed among students for better treatment and prevention of later complications.

# Literature review

- 1. **Prevalence of migraine in general and student population**

A systemic review from 1990 to 2019 concluded that migraine prevalence among the general population ranged from 2.6% to 32% (El-Metwally et al., 2020). A recent study in Malaysia suggested that the Malaysian scenario in terms of migraine profiling has not been convincing in comparison to the global picture, simply because of the lack of data from this part of the world (Rudra. et al., 2019). A descriptive cross-sectional, questionnaire-based study among the Faculty of Medicine students from first to sixth grade concluded that the prevalence of migraine was 17.9% causing moderate disability(Oraby et al., 2021). From the above literature review, it is known that the figures or percentages are subject to change between countries and races. Although it is unequally distributed among people, its impact on mental and physical health is always stronger.

# Impact of migraine and level of disability

Study results showed 89 females and 19 males with most being in the age group of 20 – 40 years had a highly significant disability and negatively impacted quality of life being affected in all domains (Pradeep R, Nemichandra S. C, Harsha S, 2020). A study conducted in Malaysia found that migraine patients with lower total quality of life scores had 1.2 times higher odds of having a disability than control. Disability was severe and frequent and was associated with lower quality of life among migraine patients (Shaik. et al., 2015). There are only a few studies that analysed the impact of migraine on disability. If the degree of disability is not recognized, one might miss the opportunity of giving effective management during attacks. Even when identified, the clinician tends to focus on treating frequency, intensity, and severity and often fails to address the impairments and disability.

# Effects of migraine on brainwaves

Electroencephalogram (EEG) is a non-invasive and relatively inexpensive method for assessing neurophysiological function of patients. A study found abnormal EEG was present in 40% of patients with migraine with aura. 24.28% of patients with migraine without aura had abnormal EEG. Slow waves most common abnormality followed by sharp and Spike waves (Sownthariya & Anandan, 2017). A study compared resting-state EEG energy intensity and effective connectivity in different migraine phases using EEG power and coherence analyses in patients with migraine without aura as compared with healthy controls (HCs). The study concluded that resting-state EEG power density and effective connectivity differ between migraine phases (Cao et al., 2016). A cross-sectional study conducted found slow waves in 10 patients (52.64%), sharp waves in 8 (42.10%), and spikes in one patient (5.26%). It has been found that 9 patients (50%) have an abnormality in the occipital region, 6 of them (33.34%) in the frontal region, and 3 of them (16.66%) in the temporal region (Hamad et al., 2014). Though the phenomenon is not yet completely understood, a study observed alpha-band hyper-synchronization in presence of visual stimuli among migraine patients (Angelini et al., 2004). A review concluded that increased alpha rhythm variability (and/or asymmetry) in the headache-free phase seems to emerge. Significant asymmetry of alpha and theta during headache has been reported in one topographic brain mapping study (Sand, 1991). Researchers at the University of Birmingham and Lancaster suggested that individuals with migraine headaches appear to have a hyper-excitable visual cortex. But there are limited studies that focus on university students with migraine symptoms indicating a need to analyze the brain activity.

# Migraine, sleep quality, and quality of life

A prospective study found that short sleep duration and low sleep quality were not temporally associated with migraine. Sleep fragmentation was associated with higher odds of migraine on day 1 (Bertisch. et al., 2020). A two-stage cluster random sampling results show 379 (14.1%) had probable migraine and 715 (26.5%) had poor sleep quality. The prevalence of poor sleep quality was 35.4% in the PM group, which was lower than that in the migraine group (47.6%), but higher than the non- headache group (21.4%) (Song et al., 2018). A cross-sectional study found that low QOL scores were associated with young ages, frequent migraine attacks, and the presence of chronic disease (AlHarbi. & AlAteeq., 2020). A study was conducted in Malaysia results showed that females with migraines had significantly lower total WHOQOL-BREF scores (84.3) than did healthy controls (91.9). The study showed that migraine sufferers experienced significantly lower QOL than the control group from a similar population (Shaik. et al., 2015). Sleep quality is an important indicator of the quality of life. Sleep disturbances are believed to contribute to the transformation from episodic migraine to chronic migraine. Quality of life is a key point in assessing the burden of disease. The above studies indicate that reduced quality of life was reported in patients with migraine when compared with age- and sex- matched people without migraine. Measuring quality of life through assessment of frequency and severity cannot exactly analysis the disease burden, instead, determination of domains through the specific instrument is important.

# Physiotherapy intervention: Aerobic exercise in migraine

In a randomized, controlled, clinical trial, participants performed aerobic exercise bike/cross- trainer/brisk walking for 45 minutes, three times/week for 3 months with an RPE 14-16 points. The results showed that aerobic exercise significantly reduced the burden of migraine and the ability to engage in physical activity (Krøll et al., 2018). A randomized control trial with High-intensity interval training (HIT), moderate continuous training (MCT), or a control group (CON) trained twice a week over a 12-week intervention period found that moderate migraine day reduction, and HIT revealed 89% likely beneficial effects compared to MCT and CON (H. Hanssen et al., 2018)**.** A three-armed randomized control trial with HIT (treadmill), MCT, or a control group (CON) trained twice a week over a 12-week intervention period found that migraine days were reduced more successfully by HIT than MCT (Henner Hanssen et al., 2017).

In a randomized control, trial participants performed outdoor fast walking for 40 minutes with a frequency of 3 times/week for 12 consecutive weeks with amitriptyline (25 mg/day) showed there was a decrease in headache frequency, intensity, duration, and depression scores (Santiago et al., 2014). In a randomized control trial, adults with migraine were trained on indoor cycling 40 minutes, 3 times/week, for 3 months with RPE 14-16 compared with control who was on daily topiramate use concluded that exercise may be an option for the prophylactic treatment of migraine (Varkey et al., 2011)**.** Office workers with neck and shoulder pain performed 10 weeks of resistance training with elastic tubing for 2 or 12 minutes per day, 5 times a week, two minutes of daily resistance training for ten weeks reduce headache frequency among office workers with neck/shoulder pain (Andersen. et al., 2011)**.** From these articles, we can conclude that aerobic exercises are effective in reducing the main characteristics like severity, frequency, duration, and intensity, but simultaneously impact of these exercises on other factors like sleep pattern, food triggers, physical activity and quality of life is limited. As of now, there is no specific protocol that is established on how aerobic exercise has to be administered to individuals diagnosed with migraine.

# Physiotherapy intervention: Biofeedback in migraine

A study combined three forms of biofeedback training: neurofeedback, breathing, and vascular with training for 4 months during which participants had 2 to 3 weeks of complete training, each training lasting for 1 hour and 30 minutes. The study concluded a reduction in the frequency of migraine attacks (Zivoder. et al., 2018). A cross-sectional study analyzed electromyography (EMG) of the neck muscles to identify whether neck pain and neck muscle tension were reported by migraine patients. Surface EMG responses of the trapezius muscle were recorded during a paradigm including rest periods, mental stress, and physical activity. The results showed that neck pain associated with migraine can therefore not be attributed to increased trapezius activity rather as an accompanying symptom of migraine (Luedtke. et al., 2018).

A 4-week study on biofeedback treatment concluded that biofeedback reduced the duration of headaches by 1.9 days, and the frequency of days when headache intensity was ≥50 by 2.4 times (Odawara et al., 2015). Electromyographic (EMG) biofeedback training for 10-50-minute sessions utilizing over frontalis and trapezius muscles and temperature from the third finger of the dominant hand concluded that biofeedback provided no additional benefit, specifically no change in the frequency or severity of the headaches compared to simple relaxation techniques ((Mullally. et al., 2009). When using EMG biofeedback, a reading of less than 2 microvolts generally indicates a fairly relaxed muscle group (G.Arena. et al., 1997). From these literature reviews, it is more evident that biofeedback is a common intervention for migraine patients. There is good evidence that biofeedback therapy can relax muscles and ease stress to reduce frequency and severity of headache, but similar studies on other parameters like sleep quality, brain activity, and quality of life are less studied indicating the need for this intervention for subjects with migraine.

# Reliability of measurement tools

Migraine Screen Questionaries’ (MS-Q): The categorical methods support the psychometric validity of the MS-Q in the study population. The clinical prevalence of migraine according to the IHS criteria was 24.7%, and 20.4% according to MS-Q. Kappa index of agreement 0.82 (p < 0.05). MS-Q sensitivity was 0.82 (95% CI, 0.81 - 0.84), specificity 0.97 (95% CI, 0.98 - 0.99) (Láinez. et al., 2005)**.**

Migraine Disability Assessment Scale (MIDAS): The test-retest reliability of the overall MIDAS score was approximately 0.8 in the US and UK. The studies described show that questionnaire is brief and easy to complete and exhibit high test-retest reliability and good validity (Ghorbani & Chitsaz, 2011).

Pittsburgh Sleep Quality Index (PSQI): A PSQI global score >5 resulted in a sensitivity of 98.7 and specificity of 84.4 as a marker for sleep disturbances in insomnia patients versus controls. The PSQI has a high test-retest reliability and a good validity for patients with primary insomnia (Backhaus et al., 2002)**.**

Migraine-Specific Quality of Life Questionnaire (MSQoL): Initial psychometric evaluation of the questionnaire indicated that it possessed adequate reliability with Cronbach's alpha for the three dimensions ranging between 0.70 to 0.85. The Questionnaire has acceptable psychometric properties and can be used to estimate the effect of migraine and its treatment on the patient's health-related quality of life (Priti hingran, Jane T.Osterhaus, David W. Miller, Jeffrey T. Lee, 2003)

Visual Aura Rating Scale (VARS): A Visual Aura Rating Scale score of 5 or more diagnosed MA with a sensitivity of 96% (95% CI 92-99%) and a specificity of 98% (95% CI 95-100%) in the derivation sample, and a sensitivity of 91% (95% CI 86-95%) and a specificity of 96% (95% CI 91- 100%) in the validation sample (Tepper, 2006)**.**

MUSE 2: Undergraduate students from the University of Victoria participated in an experiment using the MUSE portable EEG system for validation. The study concluded that improvements in the quality of low-cost portable EEG systems such as the MUSE provide an excellent opportunity for researchers to improve their ability to conduct field and/or clinical research (Krigolson et al., 2017).

1. **Methodology**

# Phase 1 study: Ethical approved

# Phase 2 Study:

- - 1. **Study design**

This study will use a randomized control trial with a pre-and post-test design that focus on the efficacy of physiotherapy intervention among university students with migraine symptoms. The independent variable will be subjects in each group (aerobic exercise, biofeedback, and control group). The dependent variable will be outcome measures which will be resting-state EEG, Pittsburgh sleep quality index and Migraine Specific Quality of life. The outcome measures will be performed at baseline, 6^th^ week, and end of intervention (12^th^ week) to compare the data obtained. This study will be carried out at UTAR Physiotherapy Center, KA Block, 3^rd^ floor (KA345) Universiti Tunku Abdul Rahman, Sungai Long Campus. Some of the instruments for intervention are to be requested from the physiotherapy center. A total of 1.5 - 2 years inclusive of baseline and end-week examination will be the estimated duration. Data collection will be after obtaining ethical approval from UTAR ethical committee. A simple random sampling method will be employed as the sampling technique in recruiting the subjects is most appropriate. Upon successful recruitment of participants, the subjects will be allocated into either of the three groups (Group 1: aerobic exercise, Group 2: biofeedback training, Group 3: control). The subjects will be given a random number in an excel sheet and ranked. Following ranking, the ranked participant will be divided by size and group accordingly.

# Participants

The target participants for this research will be undergraduate, and postgraduate students from UTAR. Sample size is estimated based on G*power statistical power analysis program software version 3.1.9.7. Using F test and statistical method as ANOVA: fixed effects, omnibus, one way with effect size of 0.49, alpha error probability 0.05, power (1-beta error probability) 0.95 and number of groups being 3, the total size estimated is 69. Then, further adjustment of sample size to accommodate 20% dropout rate (Overall et al., 2006) gives the final estimated value to 87. The participants who fulfil the following criteria will be recruited for the interventional phase. 4 of 5 on the Migraine Screen Questionnaire. 5 of 7 criteria on International Classification of Headache Disorder diagnostic criteria, 3^rd^ edition. Both genders aged between 18-40 years. The participants with a score of more than or equal to 5 on the visual aura rating scale, diagnosed to have a secondary headache, pregnancy, took medication for neurological conditions like stroke, multiple sclerosis and took medications for cardiorespiratory conditions like asthma, took medications for headache and unwilling to participate will be excluded.

# Measures

**Eligibility screening questionnaire**

The Migraine screen questionnaire (MS-Q) is a five-item migraine screening questionnaire developed for use in clinical practice. The questionnaire is based on the international headache society criteria (IHS) on migraine diagnosis (Olesen and Lipton, 1994). Each of the five items is a structured questionnaire that has an option of yes/no. A score of 0 is assigned for each “NO” response and 1 for each “YES” response. The total score is 5, where a cut-off point of ≥4 was used to indicate a case of migraine (Láinez. et al., 2005).

International Classification of Headache Disorders (ICHD) diagnostic criteria suggest at least five of seven attacks fulfilling criteria according to ICHD 3 diagnostic criteria (Society, 2021).

Visual Aura Rating Scale (VARS) is the weighted sum of the presence of five visual symptom characteristics: duration 5-60 min (3 points), develops gradually ≤5 min (2 points), scotoma (2 points), zig-zag lines (2 points), and unilateral (1 point). The maximum score is 10 points. A VARS score of 5 or more diagnosed migraine with aura (Eriksen et al., 2005).

Migraine Disability Assessment Scale (MIDAS) Questionnaire assesses headache-related disability. The respondents answer five questions, scoring the number of days, in the past 3 months of activity limitations due to migraine. 0-5 no or little disability, 6-10 mild disability, 11-20 moderate disability, and 21+ severe disability (Stewart et al., 2001).

# Instrument for assessment

A health survey questionnaire that records the general health condition of the participants and the characteristics of migraine which including the frequency, severity, and duration will be included. Level of pain scored on a four-point numerical rating scale (0–3) equivalent to no, mild, moderate, and severe pain (Olesen, 2018): 0 no pain. 1 mild pain, does not interfere with usual activities 2 moderate pain, inhibits but does not wholly prevent usual activities 3 severe pain, prevents all activities.

Pittsburgh Sleep index is a self-rated questionnaire that assesses sleep quality and disturbances over a 1-month time interval. 19 individual items generate seven component scores: subjective sleep quality, sleep latency, sleep duration, habitual sleep efficiency, sleep disturbances, use of sleeping medication, and daytime dysfunction. The sum of scores for these seven components yields one global score (Buysse et al., 1989).

Migraine Specific Quality of life is a 14-item instrument that measures the impact of migraine across three essential aspects of a patient’s health-related quality of life over the past 4 weeks: role function- restrictive (RR), role function-preventive (RP), and emotional function (EF). Raw dimension scores are computed as a sum of item response and rescaled from a 0 to 100 scale. The higher the score better is the quality of life (Rendas-Baum et al., 2013).

Recording of resting-state EEG will be performed using MUSE 2 a portable EEG recording device The headset has four dry sensors (two mastoid and two forehead sensors) and fits over the ears and extends at an angle over the middle of the forehead when properly fitted with 3 reference electrodes. The data acquisition space will be 100-200 cm from the computer/ tablet monitor displaying the stimulus presentation sequence. The participant will be instructed not to clench teeth and blink eyes often since it can alter the activity recorded. The resting-state EEG will be recorded for a total of 20 minutes alternating between eyes open and eyes closed condition (Craciun et al., 2014). Details of the experimental protocol is as illustrated in Figure 2.

Begin


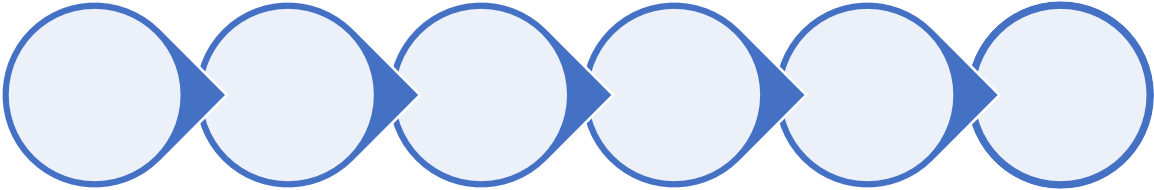

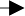

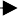


Position the participant

Electrode setup and Calibration

Eyes open

Eyes closed

Eyes open

Eyes closed

End

1 min 5 min

5 min

5 min

5 min


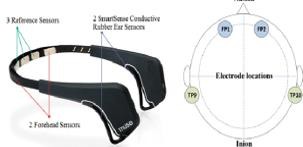

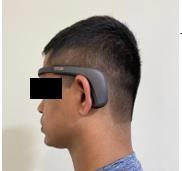

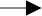

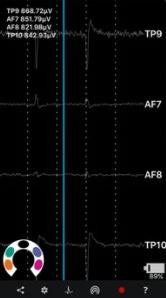

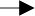


a b c

*a- MUSE 2 electrodes location, b- MUSE 2 setup, c- EEG recording from Mind Monitor App*

Figure 2: Protocol of resting-state EEG recording

# Procedure

This phase of the study will be conducted in UTAR Sungai Long campus. Figure 3 summarizes the procedure of this phase of study.


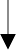

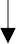

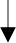


Recruitment of participants

Informed consent and Demographics

Assess for eligibility (estimated sample size 87)

**Inclusion criteria:** 4 of 5 on Migraine Screen Questionnaire, and

5 of 7 on International Headache Disorder Diagnostic Criteria

**Exclusion criteria:**

Visual Aura Rating Scale >/= 5, Secondary headache, Pregnancy, medications for neurological and cardiorespiratory diseases, medication for headache

Level of Disability assessment using Migraine Disability Assessment Scale (MIDAS)

12


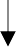

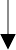


Based on level of disability using Migraine Disability Assessment Scale (MIDAS), randomization will be performed

**Pre-test**

- Health Survey
- Recording of resting-state EEG (MUSE 2 device)
- Sleep Quality (Pittsburgh sleep quality index questionnaire)
- Quality of Life (Migraine specific quality of life questionnaire)

Aerobic group (n=29) 40 minutes per session/ 36 session

Biofeedback group (n=29) 30 minutes per session/ 36 session

Control group (n=29) no intervention

At 6^th^ week and 12^th^ week intervention, **post- test**

The study will have 3 stages. Detail descriptions of each stage are as follows.

Stage 1 will be recruitment of participants. The volunteer of undergraduate and postgraduate students will be included is to confirm the migraine symptoms among students in UTAR Sungai Long campus using Migraine Screen Questionnaire and International Classification of Headache Disorders (ICHD) diagnostic criteria, additionally, these participants will be assessed for the impact of COVID-19 pandemic on migraine symptoms.

Once confirmed with migraine symptoms, Stage 2 is to analyse the level of disability using the Migraine Disability Assessment Scale questionnaire (MIDAS). The subjects in MIDAS I-IV will be randomly allocated to Group I: Aerobic exercise group, Group II: Biofeedback training group III: Control group.

In Stage 3 is physiotherapy intervention phase in which, the pre-test outcome measures will be assessed using health survey questions (level of pain, severity, frequency, and duration), the Pittsburgh Sleep index and Migraine Specific Quality of life questionnaires and recording of resting-state EEG using MUSE 2 instrument. On the first day of study, participants' migraine phases will be designated as inter- ictal, pre-ictal, ictal, or postictal based on the subjective questions. The ictal phase will be coded when the participant had suffered a migraine attack on the day of the EEG study. Pre-ictal and post-ictal phases will be coded when migraine is within 36 h before or after an ictal phase. The inter-ictal phase will be coded in a pain-free period between pre-ictal and post-ictal phases. The outcome measures will be measured at baseline, 6^th^ week, and at the end of intervention at 12^th^ week.

Following 12 weeks of intervention, recording of resting-state EEG and sleep quality using Pittsburgh sleep quality index and QoL using Migraine specific quality of life will be assessed and subjected to analyses.

Group I: Aerobic exercise training:


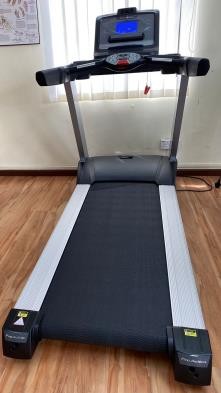

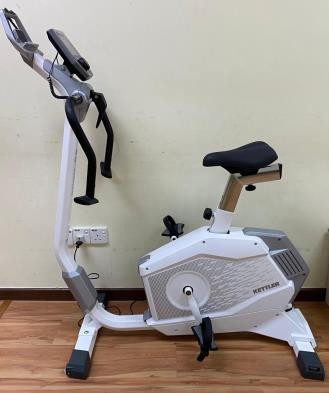
Participants in the aerobic group will undergo a training that includes walking, static bicycle, and neck exercise. The participants start the session with a warm-up for 5 minutes followed by aerobic exercise and end with a cool-down exercise for 5 minutes. 40 minutes/ session, 3 times per week for 12 weeks. During exercise, Borg’s Rating of Perceived Exertion (RPE) will be used to measure and monitor the level of physical activity. Figure 4 summarizes the exercise protocol that will be involved in aerobic exercise training group.


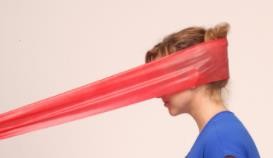

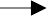

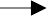


Warm up exercises (5 minutes each)

Walking (10

minutes) at 4

to 5 mph

Static Bicycle (10 minutes) pedalling speed 50 – 60 rpm

Neck exercise (10

minutes) 15 rep*

4 direction (red, 80%MVC)

Cool down exercises (5 minutes each)


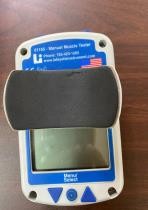

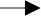

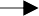
a b c

*a-Treadmill, b- Static bicycle, c- Neck exercise with hand-held dynamometer and resistance band*

Figure 4: Protocol of aerobic exercise

ProAction BH Treadmill G6700 instrument will be used for aerobic training. 75% of heart rate by age method will be used according to the participant and training will be initiated. Using Karvonen principle, the target heart rate will be calculated using the formula: [(max HR − resting HR) ×

%Intensity] + resting HR. The maximum heart rate can be calculated by 220-age. During training, the participant grips the detector, Borg’s Rating of Perceived Exertion (RPE) that measures the physical activity intensity level will be maintained at 11-14 and the speed of walking will be at 4 mph and depending on RPE response the speed will be either be maintained or increased to 5 mph.

Kettler Computeranleitung Advanced display will be used to train static cycling. Upon starting the machine, a new user items will be created, and the desired heart rate will be entered. During training, the participant grips the detector, Borg’s RPE will be maintained at 11-14. The central graphic in the display shows whether the participant is within range, or whether above or below the target range. The average exercise bike pedaling speed will be 50 to 60 rpm.

An elastic band secured around the head will be used to apply local pressure over the area. Red colour bands will be used since the target area muscle around chin or shoulder. Simultaneously, to maintain the level of resistance at 80% of the participant's maximum isometric/voluntary strength/contraction (MVC) assessed with a hand-held dynamometer as shown in Figure 4(c) during the training session. To exercise will be performed in a sitting position a single series of 15 repetitions in forward, obliquely toward the right and left, and backward.

Group II: Biofeedback training

Participants in this group will undergo a biofeedback training for trapezius and frontalis using rose for relaxation 3 times per week for 12 weeks. Each session will be for 30 minutes. Figure 5 summarizes the biofeedback training protocol.


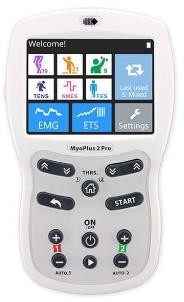

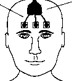

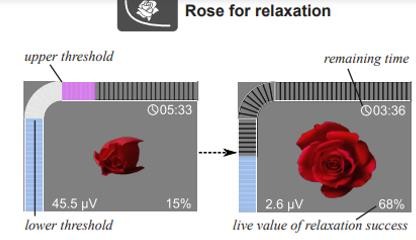

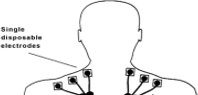

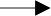

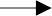


First session 5 minutes for adaptation

Electrode on Frontalis (15 minutes)

Rest time (5 minutes)

Electrode on Trapezius (15 minutes)

a b c d

*a- biofeedback training device, b- frontalis electrode placement, c- training device display/ feedback, d- trapezius electrode placement*

Figure 5: Protocol of biofeedback training

Neurotrac myoplus 4 pro instrument will be used for biofeedback training. The rose game for relaxation provides and EMG biofeedback when the participant’s rose opens when relaxation is detected, and the aim is to relax completely and stay relaxed. 4 microvolt is the default value which can still be lowered one’s relaxation ability is achieved. The electrodes will be placed on Frontalis and Trapezius muscle during each training session.

Group III: Control

The control group will receive no intervention throughout the study.

# Analysis

Analysis of EEG signals

The recorded data from MUSE 2 portable EEG device can be further analysed in MATLAB programme. The data format (CSV) using BIOSIG interface will be used to read EEG data formats in MATLAB. The sampling frequency will be set at 256Hz. To remove linear trends, high-pass filtering at 1 Hz will be done to obtain good quality ICA decompositions. Average referencing is done for source localization will be done since a non-scalp reference is used. Artifact rejection and Independent Component Analysis (ICA) will be used to remove artifacts such as muscle, eye blinks, or eye movements without removing the affected data portions.

Following extract data epochs and plot data, the EEG amplitude, and frequency, frequency band ratio, power spectrum density, coherence will be analysed. EEG will be decomposed into the five EEG sub bands of frequency and amplitude: delta (0.5–4 Hz/ 20-100 microvolt), theta (4–7 Hz/ 10 microvolt), alpha (8–12 Hz/ 2-100 microvolt), beta (13–30 Hz/ 5-10 microvolt) and gamma (30–40 Hz). For

frequency band ratio MATLAB will be used to design a filter with 'butter' command setting the cutoff frequencies according to alpha or theta band or appropriate bands and then use it on data with 'filter' command.

The power spectral density of EEG will be estimated using AR Burg method. The AR method is based on modeling the data sequence x(n) as the output of a causal and discrete filter whose input is white noise, which is expressed as the follows:


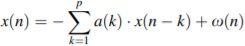


where a(k) is the AR coefficient, x(n) is the white noise of variance equal to r2, and p is the order of the AR model.

Next, the coherence estimation will be performed. Coherence represents the normalized covariance of two time series in the frequency domain. Mathematically, the coherence function Cxy(f) at a frequency f for signal x and y is obtained by the normalization of cross-spectral spectrum as follows:


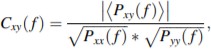


Statistical analyses

The planned statistical analysis for each phase: Data collected will be computed and analyzed using IBM Statistical Package for Social Science (SPSS) version 22.0 to produce the study results. For stages 1 and 2 descriptive analyses using mean and the standard deviation will be used to examine the data obtained. Descriptive statistics will be used to analyse the demographic data of every subject at their baseline. A box plot graph will be used to portray the distribution of data for example: age, gender, year of study, area of study and physical activity level. The scores of each outcome measure (migraine screen questionnaire, international classification of headache disorder criteria, migraine disability assessment scale and visual aura scale) will be analysed depending on scoring criteria.

For stage 3: Depending on normality of distribution, either Pearson’s or Spearman correlation test will be used to analyse the correlation between resting state EEG brain wave recording and disability. Similarly, Pearson’s or Spearman correlation test will be used to analyse the correlation between resting state EEG brain wave and characteristics of migraine. A paired t-test will be used to identify significant differences between pre-test and post-test results within same group using resting-state EEG recording, Pittsburgh sleep index and migraine specific quality of life. Furthermore, an unpaired t-test will be used to determine the post-test results between aerobic training, biofeedback and control group using the outcome measures. The Pittsburgh sleep quality index will be assessed based on domain scores of subjective sleep quality, sleep latency, sleep duration, habitual sleep efficiency, sleep disturbances, use of sleeping medication, and daytime dysfunction. The migraine sleep quality index will be assessed based on the sum of scores for these seven components yields one global score. role function-restrictive (RR), role function-preventive (RP), and emotional function (EF). Raw dimension scores are computed as a sum of item response and rescaled from a 0 to 100 scale. For resting-state EEG recording, amplitude and frequency, frequency band ratio, power spectrum density and coherence will be analysed. In phase 2, interventional phase, additionally, analysis of covariance (ANCOVA) will be used for assessing the group effect. This method combines ANOVA with linear regression on a number at different levels. The output shows the effect of the independent variable after the effects of the covariates have been removed. The independent variable will be the grouping of intervention. The dependent variable will

be the post-test measure of resting-state EEG, Pittsburgh sleep quality index, and Migraine Specific Quality of life. The covariate is the baseline measure of the dependent variable.

1. **References**

AlHarbi., F. G., & AlAteeq., M. A. (2020). Quality of life of migraine patients followed in neurology clinics in Riyadh, Saudi Arabia. *Journal of Family and Community Medicine*, *27*(1), 37–45.

Andersen., L. L., Mortensen., O. S., & Zebis., M. K. (2011). Effect of brief daily exercise on headache among adults - Secondary analysis of a randomized controlled trial. *Scandinavian Journal of Work, Environment and Health*, *37*(6), 547–550. https://doi.org/10.5271/sjweh.3170

Angelini, L., De Tommaso, M., Guido, M., Hu, K., Ivanov, P. C., Marinazzo, D., Nardulli, G., Nitti, L., Pellicoro, M., Pierro, C., & Stramaglia, S. (2004). Steady-state visual evoked potentials and phase synchronization in migraine patients. In *Physical Review Letters* (Vol. 93, Issue 3). https://doi.org/10.1103/PhysRevLett.93.038103

Backhaus, J., Junghanns, K., Broocks, A., Riemann, D., & Hohagen, F. (2002). Test-retest reliability and validity of the Pittsburgh Sleep Quality Index in primary insomnia. In *Journal of Psychosomatic Research* (Vol. 53, Issue 3, pp. 737–740). https://doi.org/10.1016/S0022-3999(02)00330-6

Bailey, L. (2017). *Brain scans show dopamine levels fall during migraine attacks*.

Bertisch., S. M., Li., W., Buettner., A., Mostofsky., E., Rueschman., M., Kaplan., E. R., Fung., J., Huntington., S., Murphy., T., Stead., C., Burstein., R., & Susan Redline, M. A. M. (2020). Nightly sleep duration, fragmentation, and quality and daily risk of migraine. *Neurology*, *94*(5).

Brandes, J. L. (2009). Migraine and functional impairment. In *CNS Drugs* (Vol. 23, Issue 12, pp. 1039–1045). https://doi.org/10.2165/11530030-000000000-00000

Buysse, D. J., Reynolds, C. F., Monk, T. H., Berman, S. R., & Kupfer, D. J. (1989). The Pittsburgh sleep quality index: A new instrument for psychiatric practice and research. In *Psychiatry Research* (Vol. 28, Issue 2, pp. 193–213). https://doi.org/10.1016/0165-1781(89)90047-4

Cao, Z., Lin, C. T., Chuang, C. H., Lai, K. L., Yang, A. C., Fuh, J. L., & Wang, S. J. (2016). Resting-state EEG power and coherence vary between migraine phases. *Journal of Headache and Pain*, *17*(1). https://doi.org/10.1186/s10194-016-0697-7

Craciun, L., Gardella, E., Alving, J., Terney, D., Mindruta, I., Zarubova, J., & Beniczky, S. (2014). How long shall we record electroencephalography? *Acta Neurologica Scandinavica*, *129*(2), e9–e11. https://doi.org/10.1111/ane.12186

Dodick, D. W. (2018). A Phase-by-Phase Review of Migraine Pathophysiology. In *Headache* (Vol. 58, pp. 4–16). https://doi.org/10.1111/head.13300

El-Metwally, A., Toivola, P., Alahmary, K., Bahkali, S., Alkhathaami, A., Al Ammar, S. A., Altamimi, I. M., Alosaimi, S. M., Jawed, M., & Almustanyir, S. (2020). The Epidemiology of Migraine Headache in Arab Countries: A Systematic Review. *Scientific World Journal*, *2020*. https://doi.org/10.1155/2020/4790254

Eriksen, M. K., Thomsen, L. L., & Olesen, J. (2005). The visual aura rating scale (VARS) for migraine aura diagnosis. *Cephalalgia*, *25*(10), 801–810. https://doi.org/10.1111/j.1468-2982.2005.00955.x

G.Arena., J., M.Bruno., G., & G.Brucks., A. (1997). The use of EMG biofeedback for the treatment of chronic tension headache. *The Biofeedbcak Foundation of Europe*.

Ghorbani, A., & Chitsaz, A. (2011). Comparison of validity and reliability of the Migraine disability assessment (MIDAS) versus headache impact test (HIT) in an Iranian population. In *Iranian journal of neurology* (Vol. 10, Issues 3–4, pp. 39–42). http://www.ncbi.nlm.nih.gov/pubmed/24250844%0Ahttp://www.pubmedcentral.nih.gov/articlerender.fcgi?artid=PMC3829228

Hamad, M. S., Sheaheed, N. M., Abdulrasool, S. M., & Almahdawi, A. M. (2014). EEG Changes in Patients with Migraine. *The Iraqi Postgraduate Medical Jouranl*, *13*(2), 156–161.

Hanssen, H., Minghetti, A., Magon, S., Rossmeissl, A., Rasenack, M., Papadopoulou, A., Klenk, C., Faude, O., Zahner, L., Sprenger, T., & Donath, L. (2018). Effects of different endurance exercise modalities on migraine days and cerebrovascular health in episodic migraineurs: A randomized controlled trial. *Scandinavian Journal of Medicine and Science in Sports*, *28*(3), 1103–1112. https://doi.org/10.1111/sms.13023

Hanssen, Henner, Minghetti, A., Magon, S., Rossmeissl, A., Papadopoulou, A., Klenk, C., Schmidt-Trucksäss, A., Faude, O., Zahner, L., Sprenger, T., & Donath, L. (2017). Superior effects of high-intensity interval training vs. moderate continuous training on arterial stiffness in episodic migraine: A randomized controlled trial. *Frontiers in Physiology*, *8*(DEC), 1–10. https://doi.org/10.3389/fphys.2017.01086

Irby., M. B., Bond., D. S., Lipton., R. B., Nicklas., B., Houle., T. T., & Penzien., D. B. (2016). Aerobic Exercise for Reducing Migraine Burden: Mechanisms, Markers, and Models of Change Processes. *Headache*, *56*(2), 357–369. https://doi.org/10.1111/head.12738.Aerobic

Krigolson, O. E., Williams, C. C., Norton, A., Hassall, C. D., & Colino, F. L. (2017). Choosing MUSE: Validation of a low-cost, portable EEG system for ERP research. *Frontiers in Neuroscience*, *11*(MAR), 1–10. https://doi.org/10.3389/fnins.2017.00109

Krøll, L. S., Hammarlund, C. S., Linde, M., Gard, G., & Jensen, R. H. (2018). The effects of aerobic exercise for persons with migraine and co-existing tension-type headache and neck pain. A randomized, controlled, clinical trial. *Cephalalgia*, *38*(12), 1805–1816. https://doi.org/10.1177/0333102417752119

Láinez., M. . J. A., Manuel Domínguez., Rejas., J., Palacios., G., Arriaza., E., Garcia-Garcia., M., & Madrigal., M. (2005). Development and Validation of the Migraine Screen Questionnaire (MS‐Q). *The Journal of Head and Face Pain*, *45*(10), 1328–1338.

Luedtke., K., Mehnert., J., & May., A. (2018). Altered muscle activity during rest and during mental or physical activity is not a trait symptom of migraine - a neck muscle EMG study. *Journal of Headache and Pain*, *19*(1). https://doi.org/10.1186/s10194-018-0851-5

Mullally., W. J., Hall., K., & Goldstein., R. (2009). Efficacy of biofeedback in the treatment of migraine and tension type headaches. *Pain Physician*, *12*(6), 1005–1011. https://doi.org/10.1111/j.1526-4610.2010.01667.x

Nayak, C. S., & Anilkumar, A. C. (2020). EEG Normal Waveforms. In *StatPearls*. https://www.ncbi.nlm.nih.gov/books/NBK539805/

Odawara, M., Hashizume, M., Yoshiuchi, K., & Tsuboi, K. (2015). Real-Time Assessment of the Effect of Biofeedback Therapy with Migraine: A Pilot Study. *International Journal of Behavioral Medicine*, *22*(6), 748–754. https://doi.org/10.1007/s12529-015-9469-z

Olesen, J. (2018). Headache Classification Committee of the International Headache Society (IHS) The International Classification of Headache Disorders, 3rd edition. *Cephalalgia*, *38*(1), 1–211. https://doi.org/10.1177/0333102417738202

Oraby, M. I., Soliman, R. H., Mahmoud, M. A., Elfar, E., & Abd ElMonem, N. A. (2021). Migraine prevalence, clinical characteristics, and health care-seeking practice in a sample of medical students in Egypt. *Egyptian Journal of Neurology, Psychiatry and Neurosurgery*, *57*(1). https://doi.org/10.1186/s41983-021-00282-8

Overall, J. E., Tonidandel, S., & Starbuck, R. R. (2006). Rule-of-thumb adjustment of sample sizes to accommodate dropouts in a two-stage analysis of repeated measurements. In *International Journal of Methods in Psychiatric Research* (Vol. 15, Issue 1, pp. 1–11). https://doi.org/10.1002/mpr.23

Pradeep R, Nemichandra S. C, Harsha S, R. K. (2020). Migraine Disability, Quality of Life, and Its Predictors. *Annals of Neurosciences*, *27*(1), 18–23. https://doi.org/10.1177/0972753120929563

Priti hingran, Jane T.Osterhaus, David W. Miller, Jeffrey T. Lee, L. K. (2003). Development and validation of the migraine-specific quality of life questionnaire. In *The Journal of Pain and Face Pain* (Vol. 38, Issue 4, pp. 295–302). https://doi.org/10.1046/j.1526-4610.1998.3804295.x

Rendas-Baum, R., Bloudek, L. M., Maglinte, G. A., & Varon, S. F. (2013). The psychometric properties of the Migraine-Specific Quality of Life Questionnaire version 2.1 (MSQ) in chronic migraine patients. *Quality of Life Research*, *22*(5), 1123–1133. https://doi.org/10.1007/s11136-012-0230-7

Rudra., T., Hasan., H. C., & Ramuni., K. (2019). Migraine in Malaysian Population_ A Cause for Alarm. *Journal of Health and Medical Research*, *1*(1), 30–31.

Sand, T. (1991). EEG in migraine: A review of the literature. In *Functional Neurology* (Vol. 6, Issue 1, pp. 7–22).

Santiago, M. D. S., Carvalho, D. de S., Gabbai, A. A., Machado, M., Pinto, P., Moutran, A. R. C., & Villa, T. R. (2014). Amitriptyline and aerobic exercise or amitriptyline alone in the treatment of chronic migraine: a randomized comparative study. *Arquivos de Neuro-Psiquiatria*, *72*(11), 851–855. https://doi.org/10.1590/0004-282X20140148

Shaik., M. M., Hassan., N. B., Tan., H. L., & Gan., S. H. (2015). Quality of life and migraine disability among female migraine patients in a tertiary hospital in malaysia. *BioMed Research International*. https://doi.org/10.1155/2015/523717

Snyder, E. J. (1990). The electroencephalogram (EEG). *Biomedical Instrumentation and Technology*, *24*(4), 296–298.

Society, I. headache. (2021). *IHS Classification ICHD- 3*.

Song, T. J., Cho, S. J., Kim, W. J., Yang, K. I., Yun, C. H., & Chu, M. K. (2018). Poor sleep quality in migraine and probable migraine: A population study. *Journal of Headache and Pain*, *19*(1). https://doi.org/10.1186/s10194-018-0887-6

Sownthariya, R., & Anandan, H. (2017). Study of EEG Abnormalities in Migraine. *International Journal of Contemporary Medical Research ISSN*, *4*(8), 2393–2915. www.ijcmr.com

Stewart, W. F., Lipton, R. B., Dowson, A. J., & Sawyer, J. (2001). Development and testing of the Migraine Disability Assessment (MIDAS) Questionnaire to assess headache-related disability. In *Neurology* (Vol. 56, Issue 6 SUPPL. 1, p. s20). https://doi.org/10.1212/wnl.56.suppl_1.s20

Taşkapilioǧlu, Ö., & Necdet, K. (2013). Assessment of Quality of Life in Migraine. *Archives of Neuro Psychiatry*, *50*(SUPPL.1), 60–64. https://doi.org/10.4274/Npa.y7310

Tepper, S. J. (2006). Eriksen MK, Thomsen LL, Olesen J. The visual aura rating scale (VARS) for migraine aura diagnosis: Commentary. In *Headache* (Vol. 46, Issue 3, p. 541). https://doi.org/10.1111/j.1526-4610.2006.00398.x

Varkey, E., Cider, Å., Carlsson, J., & Linde, M. (2011). Exercise as migraine prophylaxis: A randomized study using relaxation and topiramate as controls. *Cephalalgia*, *31*(14), 1428–1438. https://doi.org/10.1177/0333102411419681

Zivoder., I., Martic-Biocina., S., & Kosic., A. V. (2018). Biofeedback and Neurofeedback in the Treatment of Migraine. *Intech Open*.
