# Supplementary material for: Preliminary efficacy of aerobic training among university students with migraine symptoms: Study protocol for a pilot randomized controlled trial
Source: PLoS One. 2023 Sep 25;18(9):e0291534. doi: 10.1371/journal.pone.0291534 (PMC10519594; doi:10.1371/journal.pone.0291534)
Supplement: S2 File — (PDF) [file pone.0291534.s003.pdf]

We're building a better ClinicalTrials.gov. Check it out and tell us what you think!

U.S. National Library of Medicine
ClinicalTrials.gov

Physiotherapy Intervention for Migraine Symptoms

The safety and scientific validity of this study is the responsibility of the study sponsor and investigators. Listing a study does not mean it has been evaluated by the U.S. Federal Government. Know the risks and potential benefits of clinical studies and talk to your health care provider before participating. Read our disclaimer for details.

ClinicalTrials.gov Identifier: NCT05741775

Recruitment Status : Recruiting
First Posted : February 23, 2023
Last Update Posted : February 23, 2023

See **Contacts and Locations**

View this study on Beta.ClinicalTrials.gov

Sponsor:
Universiti Tunku Abdul Rahman

Information provided by (Responsible Party):
Kiruthika Selvakumar, Universiti Tunku Abdul Rahman

Study Details **Tabular View** **No Results Posted** Disclaimer How to Read a Study Record

Study Description

Go to

Brief Summary:

The goal of this randomized control trial is to analyse the effectiveness of Physiotherapy intervention among university students with migraine symptoms. The main objective is:

1. To determine the effect of aerobic exercise on the resting-state brainwaves among university students in UTAR with migraine symptoms compared with biofeedback and control exercise.
2. To analyse the influence of aerobic exercise on the sleep quality and quality of life among the cohort compared with biofeedback and control exercise.

Although the Migraine Research Foundation listed three main types of non-drug treatments for migraine are lifestyle advice, therapies, and exercises. Some common aerobic exercises such as walking, jogging, a behavioral weight loss program, cycling, and a combination of cross-training, walking, jogging, and cycling are suggested to be beneficial to the migraine patients but there remains no specific protocol established till now. Hence the other main objective of this is to establish a aerobic exercise protocol for patients with migraine symptoms.

Condition or disease	Intervention/treatment	Phase
Migraine	Other: Aerobic training<br>Other: Biofeedback Training	Early Phase 1

Detailed Description:
Habitual aerobic exercise has a major advantage of preventing or reducing symptoms of several chronic diseases and medical conditions. Aerobic or cardiovascular exercise by definition is a form of bodily movement that is fueled by aerobic energy-generating processes, where the energy demands of the exercise performed do not exceed the rate at which the cardiovascular system can supply oxygen to working muscles. Aerobic exercises have already proven to reduce frequency, duration, severity or associated disability in migraine. The physiology is that when one exercises, the body releases endorphins, which are the body's natural painkillers and natural anti-depressants chemicals called enkephalins. According to Centre of Disease Control and Prevention (CDC), adult should exercise 150 minutes of moderate intensity aerobic exercise and 2 or more days a week of muscle strengthening each week for relief of migraine or primary headaches. Similarly, a study conducted comparing neck treatment and aerobic exercise concluded that 5 weeks of intervention for migraine patient responded with better outcomes. According to American migraine foundation the exercise program, should include the cardiorespiratory fitness, flexibility exercises and muscular strengthening. For this reason, several studies encourage a multidisciplinary and tailored treatment approach for these patients. Hence the study aim to assess the efficacy of physiotherapy intervention especially aerobic exercise among university students with migraine symptoms.

Study Design

Go to

Study Type :
Interventional (Clinical Trial)

Estimated Enrollment :
87 participants

Allocation:
Randomized

Intervention Model:
Parallel Assignment

Intervention Model Description:

Three groups will be present. One group receives aerobic training, second group receives biofeedback training and the last group receives patient education. So during the trial, participants in one group receive aerobic intervention "in parallel" to participants in the other group, who receive biofeedback training and patient education.

Masking:

Single (Participant)

Masking Description:

A single blinding method will be used. The participant will not know whether he or she belongs to experimental group. Only the primary researcher performing the study will know if the participants are into aerobic training, biofeedback training or control group. Single blinding will be used in this study because the results produced are less likely to be biased.

Primary Purpose:

Treatment

Official Title:

Efficacy of Physiotherapy Intervention Among University Students With Migraine Symptoms

Actual Study Start Date :

September 18, 2022

Estimated Primary Completion Date :

September 2023

Estimated Study Completion Date :

December 2024

Resource links provided by the National Library of Medicine NIH NLM

MedlinePlus Genetics related topics: Migraine

MedlinePlus related topics: Migraine

U.S. FDA Resources

Arms and Interventions

Go to

Arm	Intervention/treatment
Experimental: Aerobic training<br>Participants in the aerobic group will undergo a training that includes walking, static bicycle, and neck exercise. The participants start the session with a warm-up for 5 minutes followed by 30 minutes of aerobic exercise and end with 5	Other: Aerobic training<br>The warm-up exercise will include light aerobic activity and some dynamic stretching movements. Similarly, the cool down exercises will include buttock stretch, hamstring stretch, inner thigh stretch, calf stretch and thigh stretch. Each stretch to be performed for 5 repetitions

Arm	Intervention/treatment
minutes of cool-down exercise. 40 minutes/ session, 3 times per week for 6 weeks.	with a hold time of 15 seconds for each. An elastic band secured around the head will be used to apply local pressure over the area. Kettler Computeranleitung Advanced display will be used to train static cycling. ProAction BH Treadmill G6700 instrument will be used for aerobic training.<br>Other Name: Group 1
Experimental: Biofeedback training<br>Participants in this group will undergo an electromyography (EMG) biofeedback training for trapezius and frontalis using rose for relaxation 3 times per week for 6 weeks. Each session will be for 30 minutes with a 5-minute resting period between each muscle session.	Other: Biofeedback Training<br>Neurotrac myoplus 4 pro instrument will be used for EMG biofeedback training.<br>Other Name: Group 2
No Intervention: Waitlist Control group<br>The control group will receive the patient education sheet with the basic information about migraine in terms of symptoms, triggers, and prevention tips. This group is also called as "waitlist control group" who will receive intervention after the active treatment group.	

Outcome Measures

Go to

Primary Outcome Measures :

1. resting-state EEG [Time Frame: 20 minutes]

   Recording of resting-state EEG will be performed using MUSE 2 a portable EEG recording device. The headset has four dry sensors (two mastoid and two forehead sensors) and fits over the ears and extends at an angle over the middle of the forehead when properly fitted with 3 reference electrodes. Once the headband is fitted, the mind monitor app will be used for data acquisition. The data obtained will be imported to MATLAB using EEGLAB function plugins (muse monitor app) CSV file. Once imported the pre-processing steps will be done to run Independent Component Analysis (ICA). Amplitude and frequency, frequency band ratio, power spectrum density and coherence will be the primary measurement analyzed for the resting-state EEG recording,

Secondary Outcome Measures :

1. Pittsburgh Sleep index [Time Frame: 10 minutes]

   Pittsburgh Sleep index is a self-rated questionnaire that assesses sleep quality and disturbances over a 1-month time interval. 19 individual items generate seven component scores: subjective sleep quality, sleep latency, sleep duration, habitual sleep efficiency, sleep disturbances, use of sleeping medication, and daytime dysfunction. The sum of scores for these seven components yields one global score. The sleep component scores are summed to yield a total score ranging from 0 to 21 with the higher total score (referred to as global score) indicating worse sleep quality.

2. Migraine Specific Quality of life [Time Frame: 10 minutes]

   Migraine Specific Quality of life is a 14-item instrument that measures the impact of migraine across three essential aspects of a patient's health-related quality of life over the past 4 weeks: role function-restrictive (RR), role function-preventive (RP), and emotional function (EF). Raw dimension scores are computed as a sum of item response and rescaled from a 0 to 100 scale. The higher the score better is the quality of life.

3. Health survey questionnaire [Time Frame: 5 minutes]

   Characteristics like frequency, severity and duration of migraine. Level of pain scored on a four-point numerical rating scale (0-3) equivalent to no, mild, moderate, and severe pain: 0 no pain. 1 mild pain, does not interfere with usual activities 2 moderate pain, inhibits but does not wholly prevent usual activities 3 severe pain, prevents all activities. The other components are expressed as either decreased/ increased/ remains the same/ unable to recall.

Eligibility Criteria

Go to

Information from the National Library of Medicine NIH NLM

Choosing to participate in a study is an important personal decision. Talk with your doctor and family members or friends about deciding to join a study. To learn more about this study, you or your doctor may contact the study research staff using the contacts provided below. For general information, Learn About Clinical Studies.

Ages Eligible for Study:
18 Years to 40 Years (Adult)

Sexes Eligible for Study:

All

Accepts Healthy Volunteers:

No

Criteria

Inclusion Criteria:

- Undergraduate, and postgraduate students from Universiti Tunku Abdul Rahman
- 4 of 5 on the Migraine Screen Questionnaire

Exclusion Criteria:

- Score of more than or equal to 5 on the visual aura rating scale,
- Diagnosed to have a secondary headache (headache attributed to the causative disorder example: infection, trauma, injury to head/ or neck)
- Pregnancy,
- Took medication for neurological conditions like stroke, multiple sclerosis and took medications for cardiorespiratory conditions like asthma,
- Took medications for headache and
- Unwilling to participate will be excluded

Contacts and Locations

Go to

Information from the National Library of Medicine

NIH NLM

To learn more about this study, you or your doctor may contact the study research staff using the contact information provided by the sponsor.

Please refer to this study by its ClinicalTrials.gov identifier (NCT number): ***NCT05741775***

Contacts

Contact: Kiruthika Selvakumar 0139441606 kiruthika@utar.edu.my

Locations

Malaysia

UTAR **Recruiting**

Kajang, Selangor, Malaysia, 43000

Contact: Kiruthika Selvakumar 0139441606 kiruthika@utar.edu.my

Principal Investigator: Kiruthika Selvakumar, MPT

Sponsors and Collaborators

Universiti Tunku Abdul Rahman

More Information

Go to

Additional Information:

The association between migraine and physical exercise.

Publications of Results:

Irby MB, Bond DS, Lipton RB, Nicklas B, Houle TT, Penzien DB. Aerobic Exercise for Reducing Migraine Burden: Mechanisms, Markers, and Models of Change Processes. Headache. 2016 Feb;56(2):357-69. doi: 10.1111/head.12738. Epub 2015 Dec 8.

Other Publications:

Santiago MD, Carvalho Dde S, Gabbai AA, Pinto MM, Moutran AR, Villa TR. Amitriptyline and aerobic exercise or amitriptyline alone in the treatment of chronic migraine: a randomized comparative study. Arq Neuropsiquiatr. 2014 Nov;72(11):851-5. doi: 10.1590/0004-282x20140148.

Varkey E, Cider A, Carlsson J, Linde M. Exercise as migraine prophylaxis: a randomized study using relaxation and topiramate as controls. Cephalalgia. 2011 Oct;31(14):1428-38. doi: 10.1177/0333102411419681. Epub 2011 Sep 2.

Wallasch TM, Kropp P. Multidisciplinary integrated headache care: a prospective 12-month follow-up observational study. J Headache Pain. 2012 Oct;13(7):521-9. doi: 10.1007/s10194-012-0469-y. Epub 2012 Jul 12.

Puledda F, Shields K. Non-Pharmacological Approaches for Migraine. Neurotherapeutics. 2018 Apr;15(2):336-345. doi: 10.1007/s13311-018-0623-6.

Parsons AA. Cortical spreading depression: its role in migraine pathogenesis and possible therapeutic intervention strategies. Curr Pain Headache Rep. 2004 Oct;8(5):410-6. doi: 10.1007/s11916-996-0016-0.

Ouyang CS, Chiang CT, Yang RC, Wu RC, Lin LC. Quantitative electroencephalogram analysis of frontal cortex functional changes in patients with migraine. Kaohsiung J Med Sci. 2020 Jul;36(7):543-551. doi: 10.1002/kjm2.12213. Epub 2020 Apr 7.

Responsible Party:

Kiruthika Selvakumar, Principle Investigator (Lecturer), Universiti Tunku Abdul Rahman

ClinicalTrials.gov Identifier:

NCT05741775 History of Changes

Other Study ID Numbers:
U/SERC/188/2022

First Posted:
February 23, 2023 Key Record Dates

Last Update Posted:
February 23, 2023

Last Verified:
February 2023

Individual Participant Data (IPD) Sharing Statement:

Plan to Share IPD:
Undecided

Plan Description:
Study protocol may be shared

Studies a U.S. FDA-regulated Drug Product:
No

Studies a U.S. FDA-regulated Device Product:
No

Keywords provided by Kiruthika Selvakumar, Universiti Tunku Abdul Rahman:
Migraine symptoms
Aerobic exercise
Biofeedback training
University students

Additional relevant MeSH terms:
Migraine Disorders
Headache Disorders, Primary
Headache Disorders
Brain Diseases
Central Nervous System Diseases
Nervous System Diseases
