# Supplementary material for: Preliminary efficacy of aerobic training among university students with migraine symptoms: Study protocol for a pilot randomized controlled trial
Source: PLoS One. 2023 Sep 25;18(9):e0291534. doi: 10.1371/journal.pone.0291534 (PMC10519594; doi:10.1371/journal.pone.0291534)
Supplement: S3 File — (DOCX) [file pone.0291534.s004.docx]

**SPIRIT SCHEDULE**

|  | **Study period** | | | | | | | | |
| --- | --- | --- | --- | --- | --- | --- | --- | --- | --- |
|  | **Enrolment** | **Allocation** | **Treatment** | | | | | | **Follow-up** |
| Timepoint | W1 | W1 | W1 | W2 | W3 | W4 | W5 | W6 |  |
| **Enrolment:**  Eligibility screen  Informed consent  Allocation | X |  |  |  |  |  |  |  |  |
|  | X |  |  |  |  |  |  |  |  |
|  |  | X |  |  |  |  |  |  |  |
| **Interventions:**  Intervention A (Aerobic Training)  Intervention B  (Biofeedback Training)  Intervention C  (Control) |  |  | X | X | X | X | X | X |  |
|  |  |  | X | X | X | X | X | X |  |
|  |  |  | X | X | X | X | X | X |  |
| **Assessments:**  Primary outcomes:   1. Resting – state EEG   Secondary outcomes   1. Characteristics of migraine 2. Sleep quality 3. Quality of life |  | X |  |  |  |  |  | X |  |
|  |  | X |  |  |  |  |  | X |  |
|  |  | X |  |  |  |  |  | X |  |
|  |  | X |  |  |  |  |  | X |  |
